# Resveratrol: a potential medication for the prevention and treatment of varicella zoster virus-induced ischemic stroke

**DOI:** 10.1186/s40001-023-01291-4

**Published:** 2023-10-05

**Authors:** Xu Wang, Hao Chen, Feiyu Song, Kuiyang Zuo, Xin Chen, Xu Zhang, Lanqian Liang, Qiyi Ta, Lin Zhang, Jinhua Li

**Affiliations:** 1 School of Public Health, Jilin University, Changchun, 130021 Jilin China; 2 College of Traditional Chinese Medicine, Changchun University of Chinese Medicine, Changchun, 130117 Jilin China; 3 China–Japan Union Hospital of Jilin University, Changchun, 130021 Jilin China; 4 Department of Neurovascular Surgery, First Hospital of Jilin University, Changchun, 130021 Jilin China; 5 Jilin Connell Pharmaceutical Co., Ltd, JilinJilin, 132013 China

**Keywords:** Varicella zoster virus, Ischemic stroke, Atherosclerosis, Resveratrol, Blood–brain barrier

## Abstract

**Background:**

Infection rate of varicella zoster virus (VZV) is 95% in humans, and VZV infection is strongly associated with ischemic stroke (IS). However, the underlying molecular mechanisms of VZV-induced IS are still unclear, and there are no effective agents to treat and prevent VZV-induced IS.

**Objective:**

By integrating bioinformatics, this study explored the interactions between VZV and IS and potential medication to treat and prevent VZV-induced IS.

**Methods:**

In this study, the VZV and IS datasets from the GEO database were used to specify the common genes. Then, bioinformatics analysis including Gene Ontology, Kyoto Encyclopedia Genes Genomes and Protein–Protein Interaction network analysis was performed. Further, the hub genes, transcription factor (TF) gene interactions, TF-miRNA co-regulatory network and potential drug were obtained. Finally, validation was performed using molecular docking and molecular dynamics simulations.

**Results:**

The potential molecular mechanisms of VZV-induced IS were studied using multiple bioinformatics tools. Ten hub genes were COL1A2, DCN, PDGFRB, ACTA2, etc. TF genes and miRNAs included JUN, FOS, CREB, BRCA1, PPARG, STAT3, miR-29, etc. A series of mechanism may be involved, such as inflammation, oxidative stress, blood–brain barrier disruption, foam cell generation and among others. Finally, we proposed resveratrol as a potential therapeutic medicine for the prevention and treatment of VZV-induced IS. Molecular docking and molecular dynamics results showed that resveratrol and hub genes exhibited strong binding score.

**Conclusions:**

Resveratrol could be an alternative for the prevention and treatment of VZV-IS. More in vivo and in vitro studies are needed in the future to fully explore the molecular mechanisms between VZV and IS and for medication development.

**Supplementary Information:**

The online version contains supplementary material available at 10.1186/s40001-023-01291-4.

## Introduction

Stroke is the leading cause of death and disability, with nearly 100 million stroke patients worldwide [1]. Ischemic strokes (IS) account for more than 80% of these patients [2]. IS has shown a trend towards lower age in recent years. Younger IS patients accounted for 31% of all stroke patients in China, and were the major source of disease burden [3]. The risk factors of strokes include hypertension, diabetes mellitus, hyperlipidemia, etc. However, youth are often missing in these risk factors. Infection has recently been identified as a major factor for IS in youth [4]. Recent studies have found a 4.5-fold increased risk of IS when varicella zoster virus (VZV) is located in the intraocular distribution of the trigeminal nerve [5].

Owing to infections, such as COVID-19, syphilis, and human immunodeficiency virus (HIV), the risk of IS have received more attention. However, the insidiousness and mild symptoms of VZV were often ignored, the most serious consequence of VZV was generally considered by the public to be postherpetic neuralgia (PHN) [6]. Although VZV-induced IS was discovered as early as 1896, the prevention of VZV-induced IS has still received less attention than COVID-19 [7]. VZV was latent after infection and reactivated when immunity was reduced. The prevalence of VZV infection is around 95%, much higher than COVID-19, the activation of latent viruses’ prevalence by VZV attacks in hospitalized patients over the age of 40 is 7.7% in China, and VZV is the only virus that replicates and produces disease in the arteries of the human brain [6, 8–10]. In the recent years, the researchers have gradually recognized the importance of preventing VZV-induced IS.

Vaccines have been introduced in many countries to prevent VZV and provide good protection against VZV-induced IS. Studies have shown that VZV vaccines can reduce the incidence of VZV by up to 70% [8]. Vaccination is an effective prevention of VZV-induced IS, especially during the high incidence of IS in the first month following VZV infection. Moreover, a single shingles vaccination can reduce this risk. However, the VZV vaccine still has some drawbacks. Most of the current varicella vaccines use the vaccine Oka, which still has the ability to infect neurons and reactivate, resulting in vaccine-induced VZV in some vaccinators [11]. In addition, some evidences have suggested that the immune response obtained from the vaccine diminishes over time [12]. In addition, the vaccine were not recommended for immunocompromised people [13]. Unfortunately, the vaccination rate of VZV vaccine has been low due to the cost and the failure of the public to recognize the importance of VZV prevention [8]. In addition, the widely used tools for detecting neurological VZV infection in IS patients are VZV DNA assay and PCR assay for VZV IgG antibody, but the accuracy of VZV assay in IS patients is not high [6]. And these tests require the patient's cerebrospinal fluid, which many patients do not accept. Thus, many patients admitted to hospital for IS have undiagnosed VZV. Despite more than 100 years of research, the molecular mechanism of VZV-induced IS has not been fully elucidated. Moreover, the vast majority of studies are reviews and case reports, and there has been little development in the mechanisms of VZV-induced IS and drug development. In addition, most importantly, there are still no effective drugs to prevent VZV-induced IS and to treat VZV-IS. Owing to the high prevalence, difficulty in diagnosis, high disease burden, and unclear mechanisms [14], the development of drugs that are safe, inexpensive, and can be taken for long-term prevention and treatment may be an effective solution to this problem. Resveratrol is a natural product that is a polyphenol with anti-inflammatory and antioxidative effects [15]. Resveratrol has the ability to cross blood−brain barrier (BBB) and the BBB permeability of resveratrol is 5.9 × 10^−6^ cm/s [16]. In addition, resveratrol has showed good protection against ischemic stroke and inhibited replication of VZV in experiments [17, 18].

Our study sought to explore the underlying mechanism of VZV-induced IS. In this study, the datasets of VZV and IS were selected from the GEO database for analysis. First, we identified differentially expressed genes (DEGs) of VZV and IS, and the common DEGs served as the basis and raw data for the whole study. Further DEGs-based analyses, including Kyoto Encyclopedia Genes Genomes (KEGG) and Gene Ontology (GO) enrichment analysis, were performed to understand the biological processes of genome-based expression studies. Subsequently, protein–protein interaction (PPI) networks were used to identify hub genes from DEGs. Further, potential therapeutic agents were searched by hub genes. Finally, we performed molecular docking and molecular dynamics (MD) simulations of potential drugs and hub genes.

## Material and methods

### Data collection and DEGs identification

All data were obtained from the GEO database. VZV data was from GSE175797, IS data was from GSE173719, GSE16561, and GSE22255. In addition, the Genecards [19] (genecards.org/) and OMIM databasesx [19] (omim.org/) were used as supplements.

**Fig. 1:**
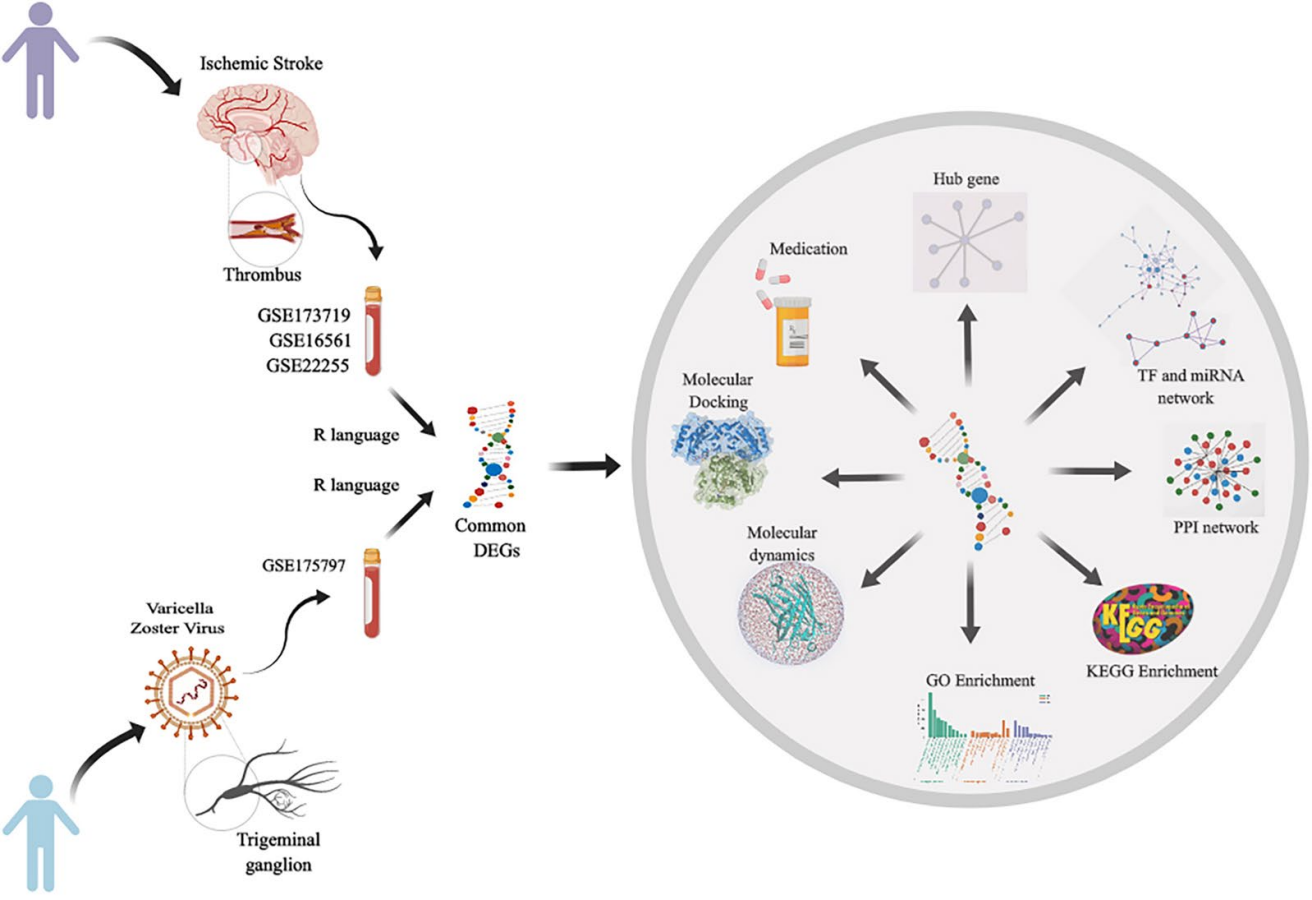
The workflow of this study. VZV and IS samples were obtained from GSE175797, GSE173719, GSE16561, and GSE22255. Common DEGs were identified from four datasets using the R language. GO identification, KEGG pathway, PPI network, hub genes, TF and miRNA analysis, and medication screening was performed based on the common DEGs. Finally, molecular docking and molecular dynamics simulations were used to validate the resveratrol and hub genes

DEGs for VZV were obtained from literature by Andrew N. Bubak et al. [21]. The processing of the IS datasets was done in the same way as that used by Andrew N. Bubak et al. Data were processed using the R project, where probes were mapped to genes and null probes were removed. If multiple probes were mapped to the same gene, a randomly selected value from the duplicate gene was used as the expression level of that gene. DEGs were identified using the LIMMA software package and adjusted for *P* < 0.05 for significantly differentially expressed genes. Owing to the small amount of sequencing data from VZV, we strictly screened the DEGs of IS to improve the accuracy of the study. Further, the Genecards database and the OMIM database were searched for "ischemic stroke" as a search term. To improve the accuracy of the obtained DEGs, we retained genes that were repeated twice as the IS targets. Finally, we compared the VZV and IS targets with the Human Protein Atlas database [22] (proteinatlas.org/) to remove the genes that were not expressed in the brain. VZV and IS intersection genes obtained from Venny website (bioinfogp.cnb.csic.es/tools/venny/). The flowchart for this study is shown in Fig. 1.

### GO and KEGG enrichment analysis

VZV-IS targets were imported into the Metascape website [23] (metascape.org/) for KEGG and GO enrichment analysis. GO and KEGG enrichment were analyzed for potential biological pathways and functions associated with the targets. *P* < 0.05 is considered that GO and KEGG pathway was significantly enrichened [24].

### Construction of the PPI and hub genes network

STRING [25] (https://string-db.org/) was used to construct a PPI network with a confidence score ≥ 0.4. The hub genes of the PPI network were identified using the Cytoscape plugin cytohubba's degree algorithm and visualized using Cytoscape (v3.7.2) [26]. The top 10 genes were considered as hub genes [27].

### TF gene interactions network

NetworkAnalyst [28] (networkanalyst.ca/) was used to find TF gene interactions with 10 hub genes.

### TF-miRNA coregulatory network

The identified hub genes were used to construct a TF-miRNA co-regulatory network using the NetworkAnalyst tool.

### ***Protein***–***chemical interactions***

An important component of this study also included the use of the Comparative Toxicogenomics Database [29] (ctdbase.com/) to identify compounds that interact with hub genes. The top 20 drugs with the highest number of therapeutic targets were compiled for analysis based on the CTD database. Parameters of each drug were analyzed using PubChem [30] (pubchem.ncbi.nlm.nih.gov), Swiss ADME (swissadme.ch/) [31], and ProTox-II database [32] (tox-new.charite.de). The aim was to find potential drugs for the prevention and treatment of VZV-induced IS.

### Molecular docking

We selected the best candidate drug suitable for the treatment of VZV-IS (Resveratrol) for molecular docking. Potential hub genes for resveratrol are used for molecular docking. The 2D structure of resveratrol was downloaded from the PubChem database and crystal structures of core targets were downloaded from RCSB protein Data Bank (rcsb.org/) [33]. Chem3D software was used to convert resveratrol into a 3D structure to minimize the energy of the structure, and then AutoDockTools 1.5.6 software was used to add hydrogen atoms to it and save it as a pdbqt file. The targets’ proteins preferably select a model with ligand binding smaller than 3 Å, and then imports into the Pymol 1.7.2 Software (pymol.org/2/) for dehydration, hydrogenation, and separation of ligands. Then crystal structures were imported into AutoDockTools 1.5.6 to construct the docking grid box for resveratrol. Docking was completed by Autodock Vina 1.1.2 software, and allowed semi-flexible docking of the molecule with the lowest binding score of resveratrol. Finally, the complexes were observed and plotted using Pymol software (such as hydrophobicity, hydrogen bonding, etc.) [34].

### MD simulation

We performed MD simulations for 100 ns according to the conformation of the optimal binding score for molecular docking. The CHARMM36 force field was used to generate the parameters of the protein [35]. Force field parameters for resveratrol are generated by the acpype.py script in AmberTools [36]. Na^+^ and Cl^−^ ions were added into the protein surface to neutralize the total charges of the systems. The resulting systems were solvated in a rectangular box of TIP3P waters extending up to minimum cutoff of 15 Å from the protein boundary [37]. The Amber ff14SB force field was employed for the protein in all of the MD simulations [38]. The system converged to a minimum energy level using the steepest descent method of 50,000 steps with a weak restraint of 10 kcal/mol force. The V-rescale temperature coupling method was used to control the simulation temperature to 300 K and the Berendsen method to control the pressure to 1 bar. Then, the equilibration process was used 100 ps of NVT (number, volume, and temperature) and NPT (number of particles, pressure, and temperature) simulations with a time step of 2 fs. In the MD simulation process, the hydrogen bonds are constrained using the LINCS algorithm with an integration step of 2 fs. Finally, a productive MD run of 100 ns was performed for all the complex systems. The MD simulations were performed with Gromacs 2019.1 [39].

## Results

### Identification of DEGs and overlap targets between IS and VZV

**Fig. 2:**
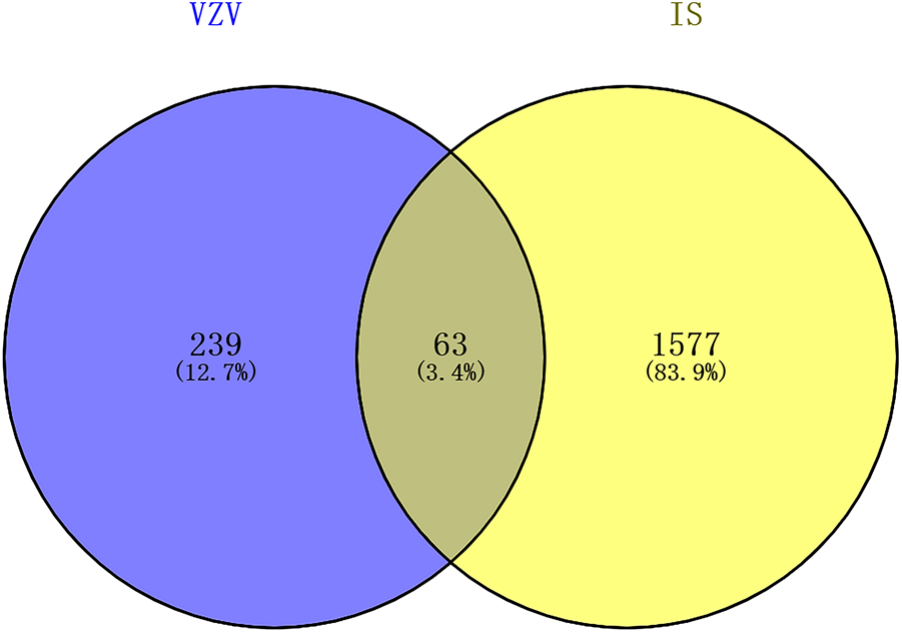
Intersection targets of VZV and IS

Finally removing the unexpressed genes in the brain, we obtained a total of 1647 IS targets and 302 VZV targets. Using Venny, 63 overlap targets were obtained and considered as VZV-IS common targets (Fig. 2).

### GO and KEGG analysis

**Fig. 3:**
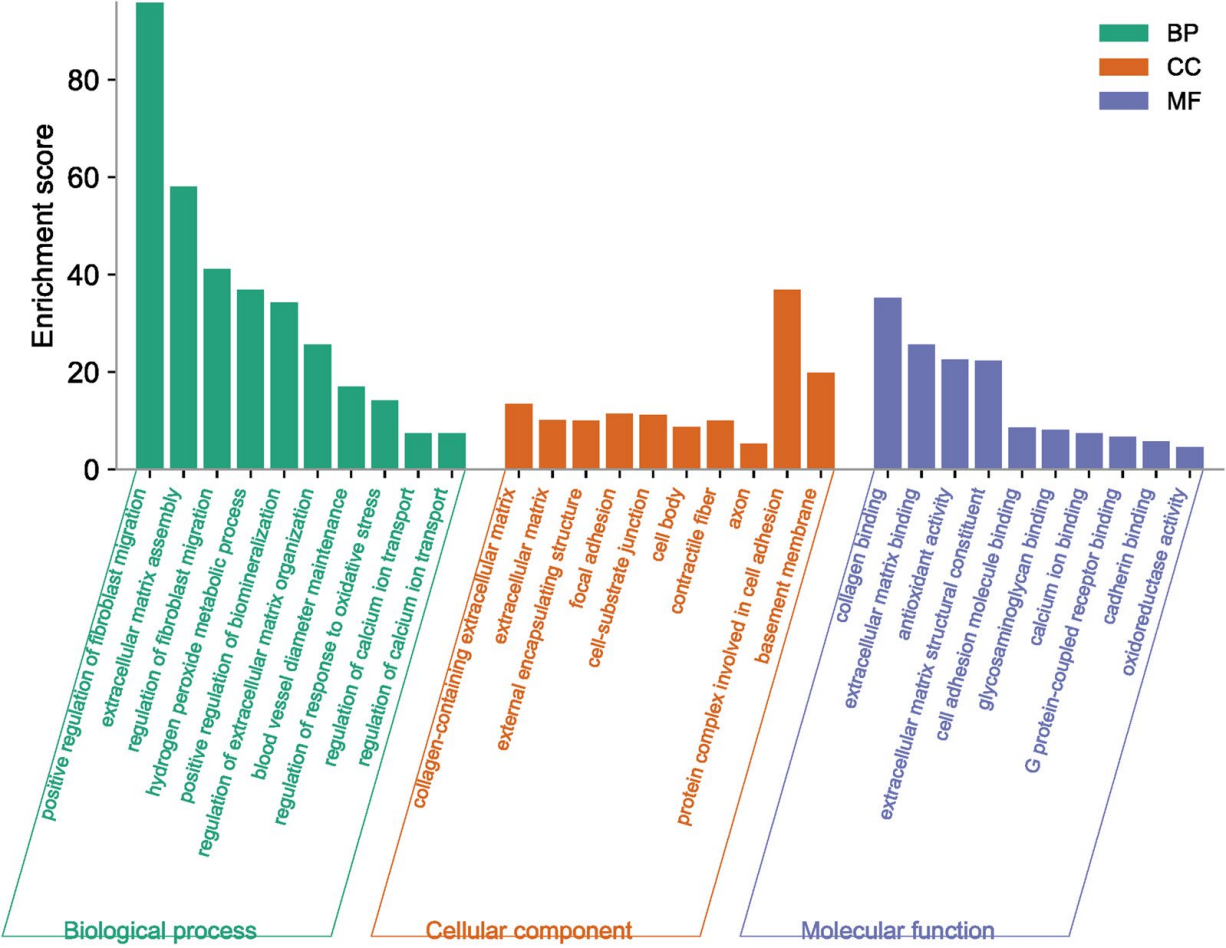
GO enrichment analysis of VZV and IS

The 63 common targets of both VZC and IS were used for GO and KEGG analysis. GO analysis included biological process (BP), molecular function (MF) and cellular component (CC). These items were shown in Fig. 3, and included (1) MF: extracellular matrix binding, cell adhesion molecule binding, etc. (2) BP: regulation of extracellular matrix organization, regulation of biomineralization, blood vessel diameter maintenance, etc. (3) CC: collagen-containing extracellular matrix, focal adhesion, basement membrane, etc.

**Fig. 4:**
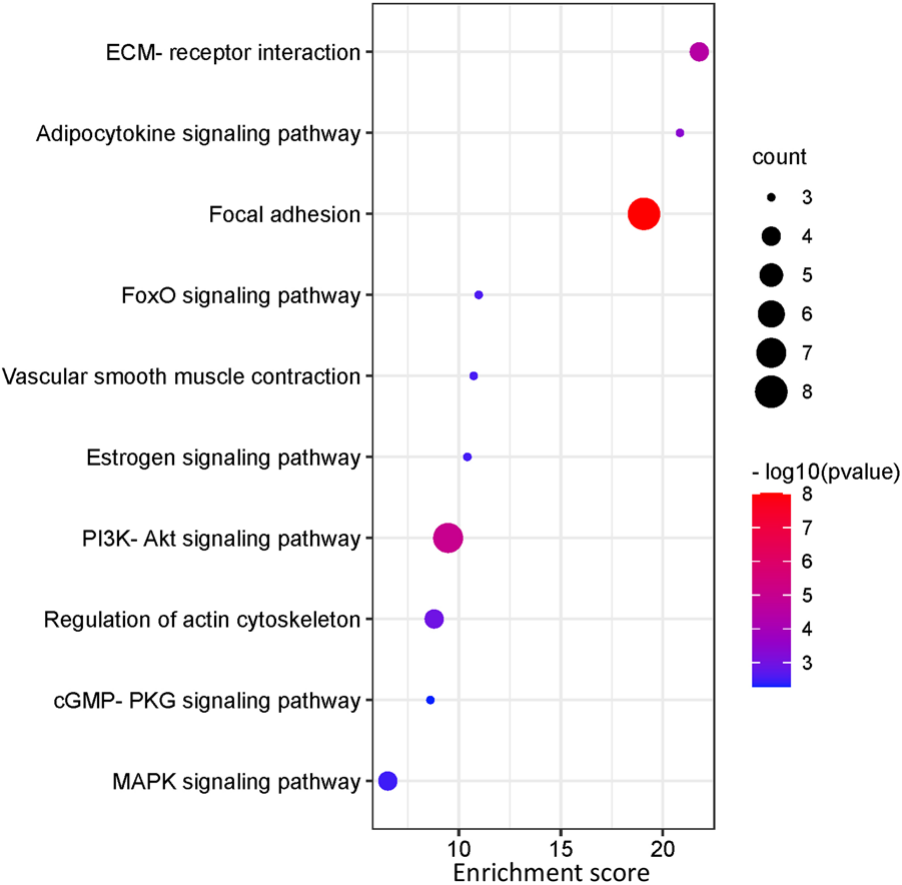
Top 10 enrichments of KEGG analysis with VZV and IS

KEGG analysis showed that these VZV-IS shared targets were enriched in focal adhesion, PI3K-Akt signaling pathway, ECM–receptor interaction, regulation of actin cytoskeleton, FoxO signaling pathway, adipocytokine signaling pathway, vascular smooth muscle contraction, estrogen signaling pathway, MAPK signaling pathway, cGMP-PKG signaling pathway, etc. (Fig. 4).

### PPI network analysis and hub genes identification

**Fig. 5:**
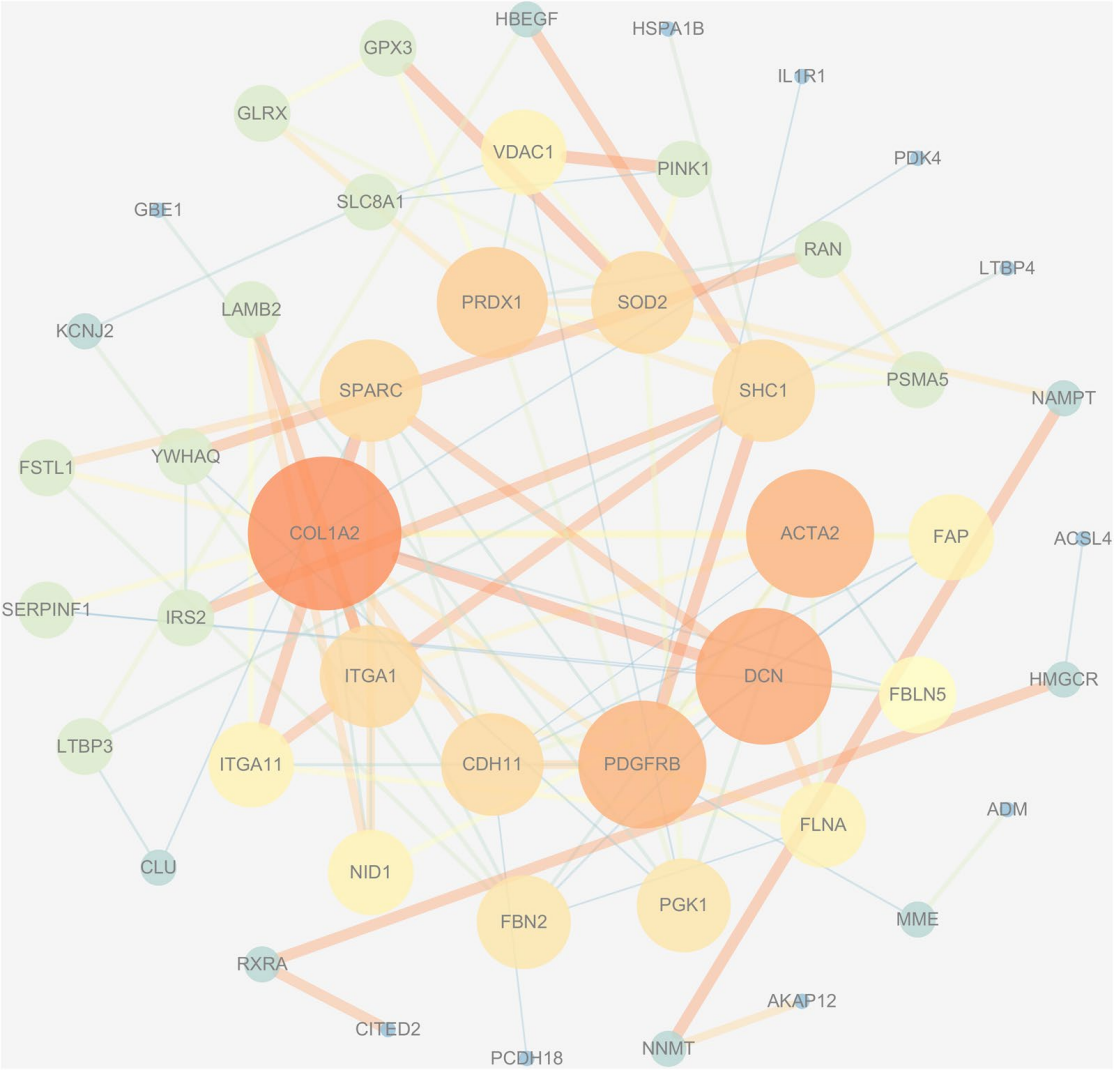
The PPI network diagram of VZV-IS targets. The nodes closer to the center and the darker color represent that they may play more important role in the whole network. The higher the degree value, the larger the area of the node, and the redder the color, the closer to the center

**Fig. 6:**
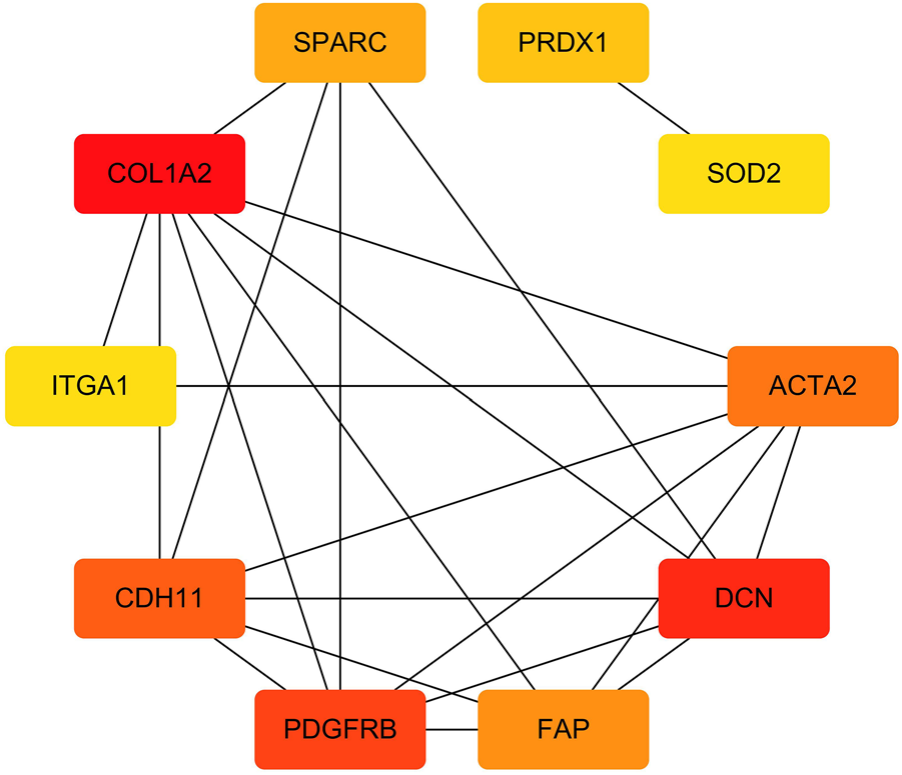
The network diagram of 10 hub genes

The PPI network includes 48 nodes (four idle nodes are removed) and 192 edges, as shown in Fig. 5. A higher degree value indicates that the node is more important in the network [40], the node is closer to the center, and the color is darker in the graph. We then used the cytohubba plug-in to identify the 10 hub genes COL1A2, DCN, PDGFRB, ACTA2, PRDX1, FAP, SOD2, SPARC, ITGA1 and CDH11 (Fig. 6). Table 1 lists the specific information and full names of the 10 hub genes, including gene symbol, protein name, and degree.

**Table 1:** The specific information of the 10 hub genes

Gene symbol	Protein name	Degree
COL1A2	Collagen alpha-2(I) chain	13
DCN	Decorin	11
PDGFRB	Platelet-derived growth factor receptor beta	10
ACTA2	Alpha-actin-2	10
PRDX1	Peroxiredoxin-1	8
FAP	Prolyl endopeptidase FAP	5
SOD2	Superoxide dismutase [Mn]	7
SPARC	Secreted protein acidic and rich in cysteine	7
ITGA1	Integrin alpha-1	7
CDH11	Cadherin-11	7

### ***TF***–***gene interactions***

**Fig. 7:**
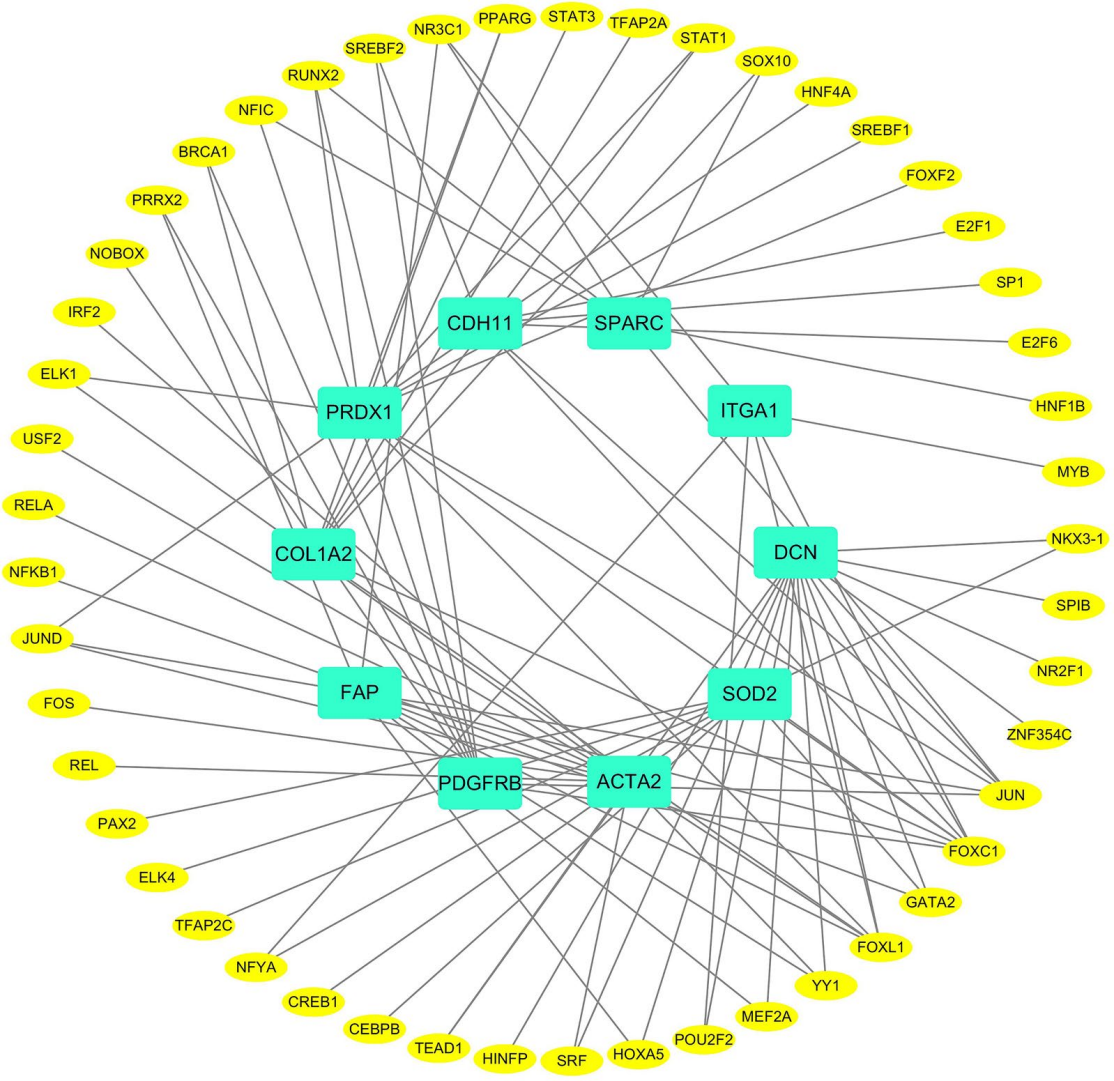
The network diagram of hub and TF genes

Ten hub genes were identified for TF genes (Fig. 7). DCN is regulated by 15 TF genes, ACTA2 is regulated by 12 TF genes, FAP is regulated by 10 TF genes, SOD2 is regulated by 9 TF genes, COL1A2 is regulated by 8 TF genes, PDGFRB is regulated by 8 TF genes, SPARC, ITGA1, CDH11 is regulated by 6 TF genes, and multiple TF genes regulate a common hub gene in the network, indicating that TF genes are closely related to hub genes and there is a high degree of interaction.

### TF-miRNA network

**Fig. 8:**
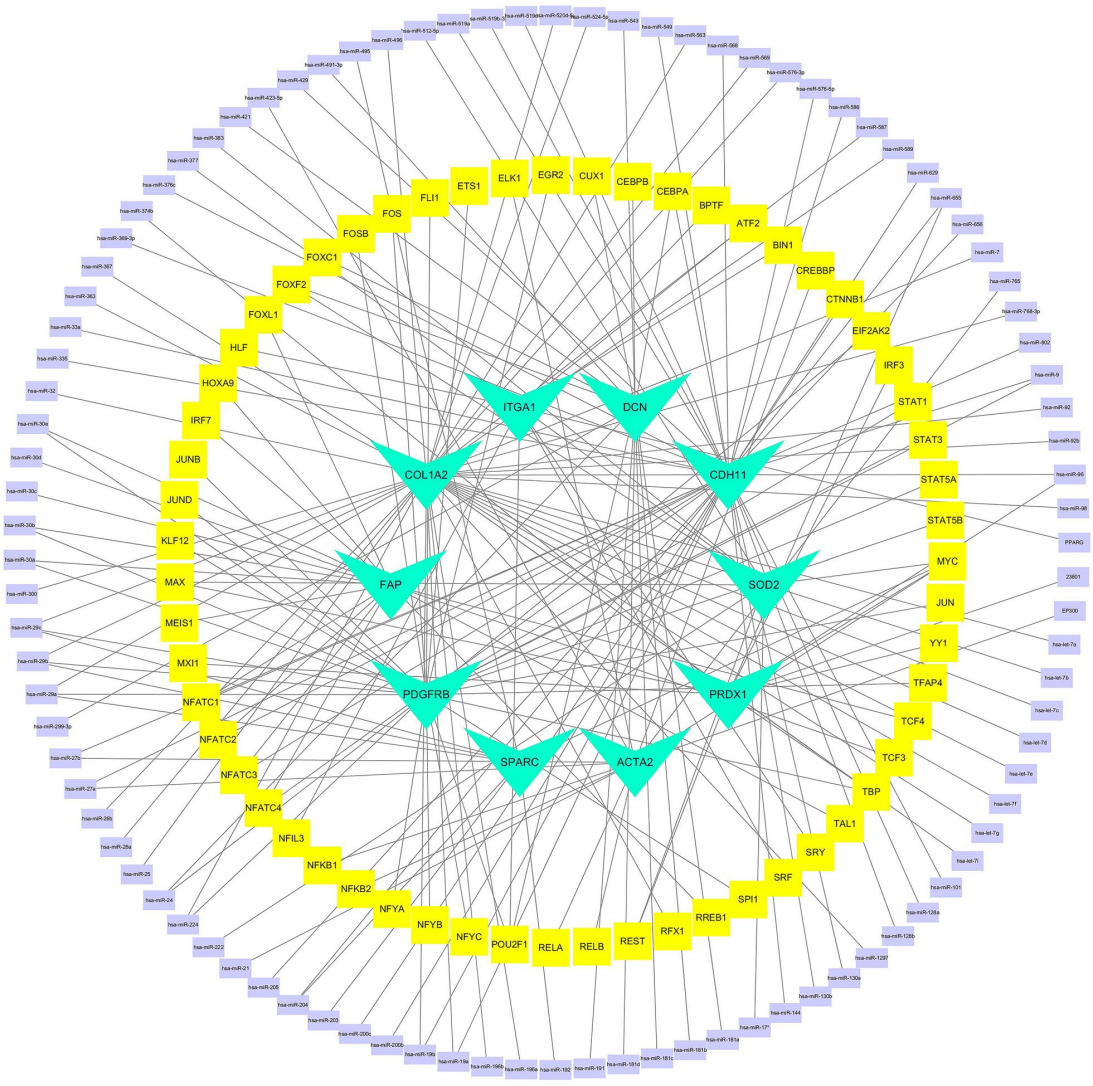
The network diagram of hub, TF genes and miRNAs

The TF-miRNA network was analyzed and provided a common interaction between miRNA and TF gene. This interaction may be involved in the regulation of hub gene expression. The TF-miRNA network consists of 166 nodes and 380 edges. 63 TF genes and 103 miRNAs formed the TF-miRNA network. The TF-miRNA co-regulatory network is shown in Fig. 8.

### Protein–chemical interactions

The specific information of 20 potential drugs for the treatment and prevention of VZV-IS is shown in Additional file 1: Table S1, including Formula, Lipinski's rule, toxicity and targets, etc. According to the characteristics of VZV-IS, preventive and therapeutic drugs should have low toxicity, small side effects, low price, and suitable for long-term use. After the final screening, resveratrol was considered to be the most promising drug. See the discussion section for specific reasons.

### Molecular docking

**Fig. 9:**
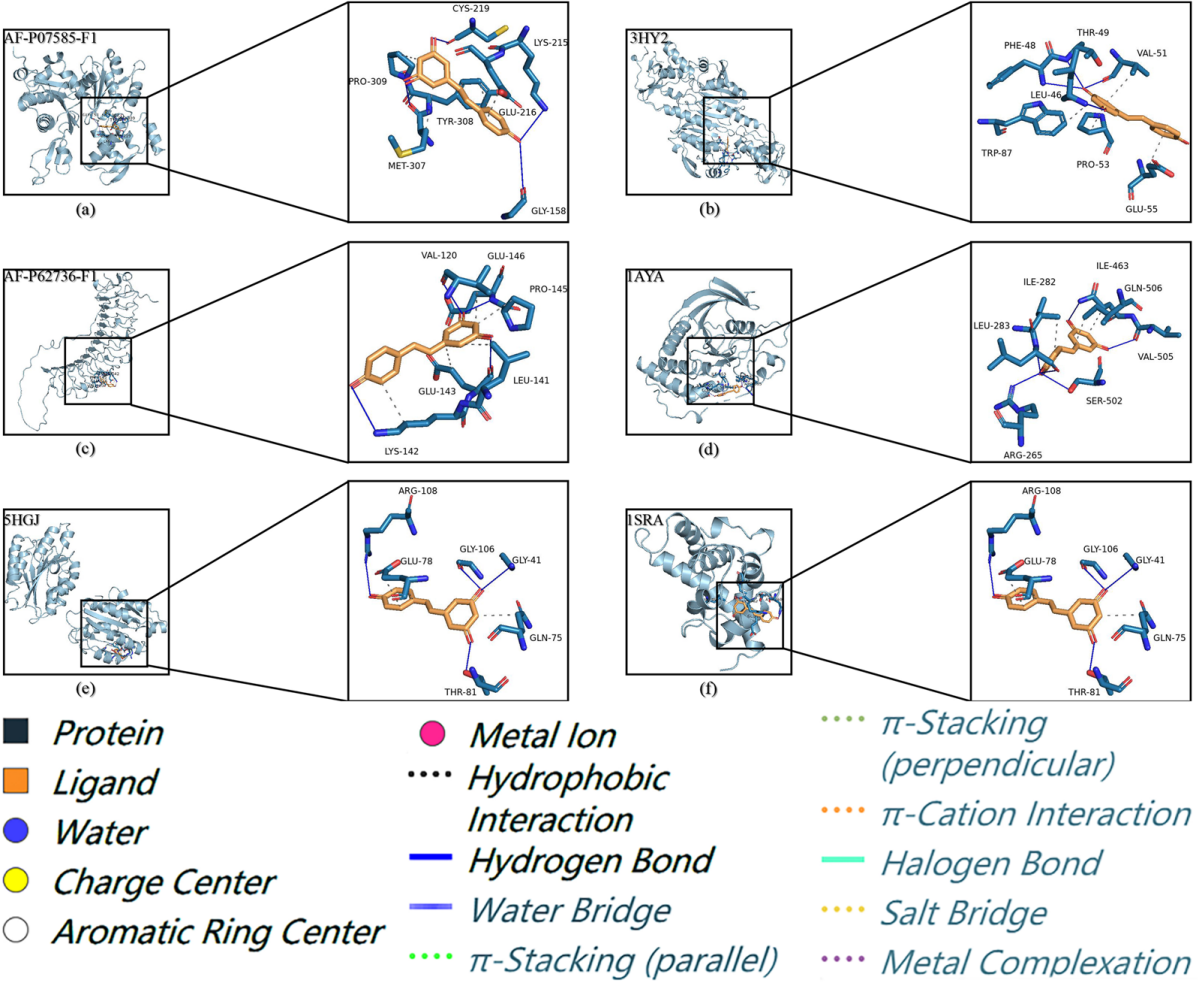
Molecular docking diagram of VZV-IS targets and resveratrol

By calculating binding score, the molecular docking results of resveratrol and targets’ proteins were predicted to be less than − 5.0 kcal/mol, indicating that these compounds had strong binding effect on key proteins. In other words, the lower the binding score of the ligand to the receptor, the more stable the binding conformation. It can be seen from Table 2 that the binding score of all docking results is lower than − 5 kcal/mol. The free binding score of the docking results ranged from − 6.3 to − 7.51 kcal/mol, indicating that resveratrol was stable in binding to the protein. The lowest binding score was found between PRDX1 and resveratrol. Binding score were attributed to hydrogen binding with LEU-46, PHE-48, THR-49, and VAL-51 residues, and hydrophobic interactions with TRP-87, PRO-53, and GLU-55. The other docking information of hydrogen bonds in Fig. 9 showed the docking situation between resveratrol and targets’ proteins. The molecular docking results for the other three drug candidates are shown in Additional file 2: Table S2.

**Table 2:** Specific information on molecular docking parameters and binding free energy

Targets	PDB/APSD ID	Box center (*x*, *y*, *z*)	Affinity/(kcal/mol)
ACTA2	AF-P62736-F1	0.225, 1.098, 0.906	− 6.88
DCN	AF-P07585-F1	− 3.941, 5.231, − 2.655	− 6.38
ITGA1	5HGJ	7.768, − 0.060, 23.447	− 6.49
PDGFRB	1AYA	23.003, 31.218, 27.749	− 6.22
PRDX1	3HY2	− 2.195, − 7.798, − 12.535	− 7.51
SPARC	1SRA	38.303, 17.827, 30.274	− 6.30

### MD simulation

**Fig. 10:**
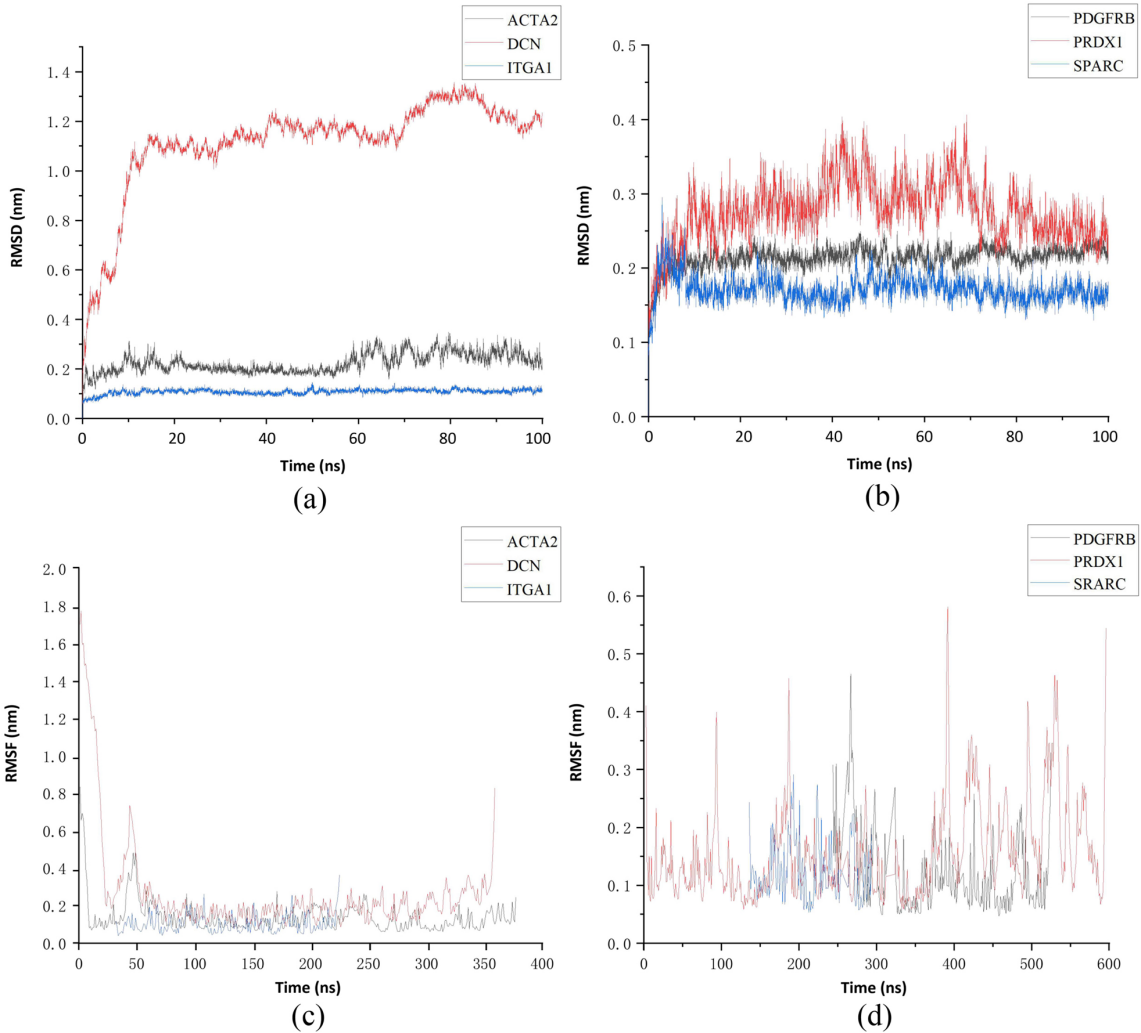
Profiles of molecular dynamics simulations between target proteins and resveratrol. **a** RMSD of resveratrol–ACTA2 complex, RMSD of resveratrol–DCN complex and RMSD of resveratrol–ITGA1 complex. **b** RMSD of resveratrol–PDGFRB complex, RMSD of resveratrol–PRDX1 complex, and RMSD of resveratrol–SPARC complex. **c** RMSF of resveratrol–ACTA2 complex, RMSF of resveratrol–DCN complex, and RMSF of resveratrol–ITGA1 complex. **d** RMSF of resveratrol–PDGFRB complex, RMSF of resveratrol–PRDX1 complex, and RMSF of resveratrol–PRDX1 complex

To further study the interaction between resveratrol and the targets, we used MD to simulate the protein–resveratrol complex for 100 ns. The root-mean-square deviation (RMSD) from the average structure of backbone atoms for each MD trajectory was calculated as well for exploring the "position stability" for each complex. Figure 10A, B was plotted the RMSD of backbone atoms of the complex system and the result showed that after 12 ns, the conformation of all systems has reached a steady-state because the RMSD value fluctuates for the original structure of complex within 0.2 nm which indicates the stability of the structures. As shown in Fig. 10C, D, the root mean square fluctuation (RMSF) has provided details about the structural flexibility of individual residues in a protein. The fluctuations of these systems in a small region were relatively high. On the contrary, most of the residues had low fluctuation values in other regions, which indicated that the residues are stable in binding to the protein. The above results indicated that the resveratrol is structurally stable with these proteins in MD simulations.

## Discussion

VZV infection is a major risk factor for IS, especially in young adults without underlying disease [41, 42]. Previous studies have suggested that VZV causes IS by the following process: VZV virus infects cerebral arteries, causing acute endothelial damage, and chronic inflammation promotes cytokine secretion, leukocyte recruitment, and vascular smooth muscle cell proliferation, inducing atherosclerotic plaque formation. And the risk factors for atherosclerosis (i.e. diabetes mellitus, hypertension, and hyperlipidemia) exacerbate plaque formation. IS is the final event, which is caused by plaque rupture and thrombus block vessels. However, the process of plaque formation is still unknown. This study was conducted with a view to exploring the mechanism of VZV leading to IS and its potential therapeutic agents through bioinformatics. Firstly, 63 DEGs were identified in the VZV and IS datasets, followed by KEGG, GO and PPI network analysis of the 63 DEGs. Subsequently, 10 hub genes were identified, including COL1A2, DCN, PDGFRB, ACTA2, PRDX1, FAP, SOD2, SPARC, ITGA1, and CDH11. Further, TF-gene interactions, TF-miRNA co-regulatory network and drug candidate analysis were performed by hub genes. Finally, the potential drug (resveratrol) were performed by molecular docking and MD simulations to the disease targets.

The ten hub genes are strongly associated with the occurrence and development of IS. (1) Heterozygous COL1A2 were found to be associated with IS [43]. COL1A2 is associated with cranial vascular integrity and can lead to vascular malformations. COL1A2 affects the function of collagen and perivascular fibroblasts in vascular stabilization [44]. COL1A2 polymorphism destabilizes collagen protofibrils in a Chinese population [45]. (2) DCN is a small leucine-rich proteoglycan that mediates the formation, organization and tensile strength of collagen fibers. In abdominal aortic aneurysm, DCN is reduced in the extravascular membrane, leading to vessel wall instability and consequent vessel rupture. CAR-DCN treatment increases DCN and collagen levels in the vessel wall [46]. DCN expression correlated with IS severity, and DCN expression was significantly lower in patients with IS [47]. (3) PDGFRB plays an important role in vascular development by promoting the proliferation of pericytes and smooth muscle cells to endothelial cells, neointima formation at sites of vascular injury, and contributes to the rearrangement of the actin cytoskeleton. Recent studies have found PDGFRB to be closely associated with thrombosis and IS [48]. Pericytes may enhance peri-infarct oligodendrocyte formation and astrocyte proliferation to promote intra-infarct fibrotic repair after IS, possibly mediated by PDGFRB [49]. (4) ACTA2 encodes smooth muscle actin and is involved in vasoconstriction and blood pressure homeostasis. Mutations in this gene could cause a variety of vascular diseases including IS. ACTA2 triggers cerebral artery disease with abnormal internal carotid circulation coupled with proximal segmental dilatation, distal segmental occlusive disease and prolonged dilatation, predisposes children to IS [50]. Vascular pathology analysis of smooth muscle cells and myofibroblasts extracted from patients with ACTA2 mutations showed that increased proliferation of smooth muscle cells led to occlusive disease, indicating that ACTA2 mutations predispose to early-onset of IS [51]. (5) PRDX1 is a stress-inducible macrophage redox protein that plays a role in cellular resistance to oxidative stress and may contribute to the antiviral activity of CD8(+) T cells. PRDX1-dependent antioxidant microglia increase transcriptional levels of protective molecules in IS [52]. PRDX1 is involved in inflammation and cellular injury in IS by interacting with TLR4 [53]. Previous studies found a protective effect of PRDX1 against endothelial hyperactivation and atherosclerosis [54]. The recent studies have found that PRDX1 levels are positively correlated with the severity of IS and the severity of prognosis of patients [55]. (6) FAP is involved in the control of epithelial–mesenchymal interactions in the process of fibroblast growth or development, and tissue repair. The recent studies have found that patients with reduced FAP activity have more severe IS and worse short-term prognosis [56]. (7) SOD2 has a protective effect against oxidative stress and endothelial dysfunction in carotid arteries [57]. It was found that SOD2 overexpressing mice had a reduced chance of bleeding, suggesting that SOD2 has a protective effect on vascular integrity [58]. (8) SPARC plays important functions in the central nervous system, such as synapse stabilization and axonal regeneration, and is associated with IS severity [58]. SPARC induces angiogenesis after cortical injury [59], and SPARC expression is increased in patients with atherosclerosis and calcified plaques [60]. Recent studies have found that SPARC deficiency reduces the inflammatory response in IS and increases the integrity of BBB [61]. (9) ITGA1 may control the exit or persistence of macrophages in inflamed tissues to regulate the inflammatory response [62]. Knockdown of ITGA1 reduces inflammation and angiogenesis [63]. (10) CDH11 has the ability to regulate collagen and elastin, affecting the mechanical properties and contractile function of blood vessels [64], and its expression is upregulated in vascular calcification diseases [65]. Inhibition of the CDH11 signaling pathway attenuates the migration and proliferation of vascular smooth muscle, which is a pathological hallmark of endothelial proliferation [66]. CDH11 is required for the differentiation of mesenchymal stem cells into smooth muscle cells and affects the contractile function of blood vessels [67]. Recent studies have found that CDH11 is associated with vascular malformations [68]. CDH11 is critical in the pathology of IS. These above evidences suggest that 10 hub genes are important in VZV-IS. This suggests these hub genes were closely associated with the pathological process and prognosis of IS, and the changes in these hub genes were involved in the development of IS after VZV activation.

Furthermore, regulatory biomolecules are potential biomarkers for a variety of diseases, including IS. The miRNA and TF genes of hub gene were used to analyze the regulatory network, and a total of 103 miRNAs and 63 TF genes were identified. Among the TF genes with the strongest interaction, the degree values were JUN, FOS, CREB, BRCA1, PPARG, STAT3, JUND, SRF, FOXC1, Sox10, and ELK1. These TF genes also play an important role in IS. JUN plays an important role in neurodegeneration, cell death and repair after IS [69]. CREB is a transcription factor that plays a key role in neuronal excitability, improves motor recovery after IS, and prevents recurrence of IS [70]. BRCA1 is a tumor suppressor, and a recent study found that overexpression of BRCA1 reduced reactive oxygen species production and lipid peroxidation after IS to increase DNA repair [71]. Recent studies have found that PPARG polymorphisms may be an independent risk factor for IS [72]. STAT3 can treat IS through several mechanisms [73]. JUND can inhibit IS inflammation [74]. SRF acts as a major transcriptional regulator of vascular endothelial junction stability and ensures the physiological function of the cerebrovascular system [75]. FOXC1 is known to regulate skull and brain developmental processes and can modulate inflammatory responses after IS [76]. Sox10 is a transcription factor that plays a central role in glial cell development and maturation, enhancing oligodendrocyte production and white matter repair [77]. ELK1 is related to inflammatory response, endothelial dysfunction and atherosclerosis in IS [77, 78]. The highest miRNA degree value was miR-29. Previous studies have found that arterial wall tissue injury may be related to up-regulation of miR-29 expression [79]. This suggested that these miRNA and TF genes are also extensively involved in the development of IS.

We further revealed more complex pathological processes based on GO and KEGG enrichment analysis of DEGs of VZV-IS and other findings. The GO and KEGG enrichment results can be mainly classified into the following categories: (1) inflammation and oxidative stress response, (2) regulation of vascular morphology and function, (3) regulation of extracellular matrix (ECM), (4) regulation of ion transport, and (5) regulation of cell adhesion. It is suggested that there are extremely complex regulatory mechanisms of VZV-induced IS. We have addressed these possible mechanisms.

Inflammation and oxidative stress are the initial pathological processes after VZV activation. Multiple GO and KEGG items suggested that inflammation and oxidative stress played an important role in the process of VZV-IS, including antioxidant activity, oxidoreductase activity, hydrogen peroxide metabolic process, PI3K–Akt signaling pathway, FoxO signaling pathway, MAPK signaling pathway, cGMP–PKG signaling pathway, etc. VZV is first activated in the outer arterial membrane and early on there are neutrophils involved in the immune response, generating reactive oxygen species, leading to an imbalance in oxidative stress, inducing apoptosis, and loss of vascular smooth muscle cells [80–82]. Over time, VZV gradually invades the mid and inner membranes. Cell adhesion and migration is also an important factor in the process of VZV leading to atherosclerosis. When endothelial cells are activated, many cells adhere and migrate, and release cytokines involved in binding to endothelial cells and penetrating the arterial wall to exacerbate inflammation.

Subsequently, low-density lipoprotein (LDL) undergoes oxidation and accumulates in the vessel lining, and monocytes differentiate into macrophages that phagocytose oxidized LDL deposits and transform into foam cells, leading to the formation of atherosclerotic plaques [83]. In the late stage of atherosclerosis, a large number of inflammatory cytokines infiltrate the vessel wall, and the synergistic effect of all pro-inflammatory signals within the plaque not only enhances inflammation but also secretes matrix metalloproteinases (MMPs). MMPs could degrade collagen fibers in the ECM of the plaque and impede the mechanical stability of inflamed tissue to support renewal of structural elements [84], leading to plaque rupture, hemorrhage, and thrombosis. The enrichment results of GO and KEGG include Focal adhesion, cell adhesion molecule binding, glycosaminoglycan binding, calcium ion binding, G protein-coupled receptor binding, cadherin binding, positive regulation of fibroblast migration and other processes. These results indicate that the cell adhesion and migration are involved in the pathological process of arteriosclerosis.

The ECM is an important component of the BBB structure. Immune thrombosis in COVID-19 is dominated by pulmonary venous and microvascular thrombosis [85–87]. VZV differs from COVID-19 in that directly infects cerebral arteries, unlike other parts of the vasculature, an important barrier exists in the blood vessels within the brain that is the BBB. ECM disrupted by MMPs, which leads to increased BBB permeability, further allows peripheral immune cells to enter the endothelium, exacerbating the inflammatory response and oxidative stress. Severe cases can trigger hemorrhagic transformation, often with much more severe consequences than IS. Exacerbated cellular inflammation secretes cytokines that further promote vascular smooth muscle death, leading to intimal thickening with myofibroblast accumulation. The whole process enters a vicious cycle. Results of KEGG and GO enrichment analysis are widely enriched in ECM–receptor interaction, regulation of actin cytoskeleton, ECM binding, ECM structural constituent, collagen-containing ECM, focal adhesion, protein complex involved in cell adhesion, basement membrane, and other items. This suggests that the disruption of the ECM is involved in the VZV-IS pathological process.

Over time, vascular calcifications can occur in the intima or mesoderm of the arterial wall, increasing arterial stiffness [88]. Calcification is usually considered beneficial in that it makes the plaque stable, but this also correlates with the nature of the plaque, spotty calcifications on the contrary, in other words the calcification effect of the plaque is bidirectional [89]. However, the outcome of this VZV-induced plaque calcification may be harmful in terms of the increased risk of IS within one year of VZV activation. As plaque enlarges and the elastic layer disrupts, it eventually leads to atherosclerosis. There are no studies focusing on atherosclerosis due to VZV. Determining the mechanism of plaque calcification due to VZV will need to be confirmed in future studies. Several items in GO enrichment are related to biomineralization, including calcium ion binding, cadherin binding, regulation of calcium ion transport, positive regulation of biomineralization, etc. This suggests that atherosclerosis due to biomineralization is involved in the pathological process of VZV-IS.

KEGG and GO enrichment analysis are enriched in vascular smooth muscle contraction, vasculogenesis, blood vessel diameter maintenance, cGMP-PKG signaling pathway, and other items. This suggests that nitric oxide (NO) is also an important pathological process involved in VZV-IS. The suppression of VZV immunity that may result from NO production during inflammation is mediated by T cells [90]. Regulation of actin cytoskeleton and NO are closely related, and these mechanisms may reduce vascular resistance by increasing secretion of MMPs to depolymerize actin [91, 92]. However, this is associated with the accumulation of more damaging ECM by MMPs released through inflammation and oxidative stress, which exacerbates BBB and vascular structural damage and causes more severe inflammation. Not only inflammatory and immune responses, but also vascular regulation receives regulation by NO, such as contraction of vascular smooth muscle. Human defense regulatory mechanisms may play a deleterious role in the VZV-IS process. Contraction of vascular smooth muscle narrows the internal diameter of the vessel, increasing the flow rate and exacerbating atherosclerosis with destruction of elastic and collagen fibers, and rupture of the internal elastic layer. Sclerotic arteries and plaques reduce the elasticity and diameter of arteries, which in turn causes a decrease in blood flow rate. To maintain blood flow rate, vascular smooth muscle contraction causes an increase in blood pressure, and high blood pressure further leads to atherosclerosis. Atherosclerosis eventually leads to complete blockage of the vessel causing IS in two ways, including plaque rupture and nonrupture. Atherosclerosis without rupture leads to vascular occlusion due to atherosclerotic plaques that continue to expand until they completely block the vessel, which does not need to be elaborated. When atherosclerotic plaque ruptures, it leads to platelet aggregation, thrombosis, and vascular occlusion. In pathophysiology, platelets and activated endothelial cells can be associated with the secretion of protein disulfide isomerases. Protein disulfide isomerases can react with free radicals and promote thrombosis. Healthy arterial endothelial cells limit clot formation by activating eNOS to release NO, thereby controlling clot size. Endothelial cells can also limit thrombus formation by blocking platelet activation through the release of adenosine and acting as anti-inflammatory mediators through their receptors [93]. Owing to impaired function, NO release is inhibited and endothelial cells located around atherosclerotic lesions lose their ability to regulate clot propagation [94, 95]. Several other factors exacerbate this process, including estrogen, LDL, and calcium ions.

Estrogen pathway was found in KEGG enrichment of VZV-IS. We suggest that the estrogen protection against VZV in women is bidirectional. Studies have shown that the risk of VZV-induced atherosclerosis is higher in men than in women [82]. 50.7% of VZV infections in men occurred before 45 years of age, but only 23.5% in women, while in the total population, infections were more common in women than in men, suggesting that postmenopausal women with abrupt changes in estrogen levels are exposed to VZV [96]. It is clear that estrogen plays a crucial role in the pathogenesis of VZV-IS, although this mechanism is not clear. Because the protective effects of estrogen are broad, including positive effects on blood pressure, lipids, and glucose, it is noteworthy that estrogen is equally protective against IS. Studies have found that the expression of calcium-related genes and intracellular calcium content are increased after VZV infection of cells [97].

GO enrichment results were enriched in calcium ion binding, regulation of calcium ion transport, regulation of calcium ion transport, and other items. It is suggested that the mechanism of VZV leading to atherosclerosis may be related to the regulation of calcium. Unlike the normal slow progression of atherosclerotic plaques, atherosclerosis due to infection is rapid and unstable. In particular, VZV directly invades the outer membrane of the cerebral arteries and then the inner membrane leading to atherosclerosis and stenosis within the cerebral arteries. In addition, due to the intense inflammation and immune response, this process is more rapid and intense than normal atherosclerotic plaque formation. Therefore, the year of VZV outbreak is a high-risk period for IS.

Hyperlipidemia is one of the risk factors for VZV-induced IS. However, the study found that the lipid-lowering drug statin increased the risk of VZV development, a result that is clearly confusing [98, 99]. This may be related to immunothrombosis. The response to blood-borne pathogens and tissue damage is a coordinated intravascular coagulation, recently termed immunothrombosis, which allows platelets and immune cells to form a physical barrier that prevents pathogen transmission and activates the immune system. In contrast, platelets carry transcripts of all pathogen-sensitive toll-like receptors. During certain bacterial infections, platelets are able to induce prothrombotic events, secrete cytokines, chemokines and antimicrobial peptides, leading to bacterial isolation and destruction [100]. It is unclear whether thrombosis in viral infections is defense system reactive or similar to the function during bacterial infections. Immunothrombosis is mainly associated with neutrophils, platelets, coagulation factors, fibrinogen and monocytes [101]. The main process is such that endothelial activation causes platelet and leukocyte attachment due to the activation of inflammatory venous endothelium and increased expression of surface selectins. The attached leukocytes are activated and initiate the expression of tissue factor, which in turn activates the coagulation cascade. The protective anticoagulant effect of the endothelial surface is counteracted by low blood flow. Low blood flow may lead to hypoxic conditions, which have been described to increase the expression of endothelial adhesion molecules and the consequent leukocyte adhesion that occurs. Therefore, the treatment of atherothrombosis includes prevention of platelet activation and aggregation, and prevention of atherosclerotic plaque rupture. According to common sense statin instead has a plaque stabilizing effect that protects against VZV-IS. However, on the contrary, statin increases the spread of VZV, and this is puzzling.

We propose a possible mechanism by which the body's defense mechanisms are activated after VZV infection, inducing plaque to accumulate at the VZV-infected site, form plaques that wrap around the infected area, and prevent VZV transmission. The increased risk of VZV development by statin treatment may be due to that it is the body's defense mechanisms that induce the conversion of high-density lipoprotein cholesterol (HDL-C) to LDL, and statin treatment causes a lack of raw material for the formation of plaques covering the infected area, leading to an increased risk of plaque shedding. This also exposes a drawback of most drugs that they are too homogeneous in their therapeutic mechanisms, which is a fatal drawback in complex diseases. It suggests that we should adopt a multi-targeted therapy for the treatment and prevention of VZV-IS.

The treatment of VZV-IS is now mainly based on the antiviral, hormonal, and symptomatic therapies [102, 103]. There are some potential risks associated with these treatments, for example: hormonal therapy has been found to be associated with an increased risk of stroke in the first years of treatment [100]. A recent cross-sectional study of 2,787 postmenopausal women receiving hormone therapy suggested that both estradiol and sex hormones promoted prothrombotic events [104]. The complex pathological mechanism of VZV-IS makes it difficult to study its specific molecular mechanism in a short time. However, it is urgent to develop a safe drug with few side effects and adverse reactions to protect VZV patients from developing IS as soon as possible.

We screened 20 drugs based on the hub genes of VZV-IS. However, as a drug to protect or prevent VZV-induced IS, it should have several characteristics: (1) conform to Lipinski's rule of five, (2) have good bioavailability, (3) be able to cross the BBB, (4) have low toxicity, (5) be affordable, (6) be multitarget therapeutic, (7) have low adverse effects when used with other drugs, and (8) preferably already in wide use. Resveratrol, Melatonin, Menthol, and Aspirin conform all these characteristics.

Resveratrol is the best choice among these four alternative drugs. Resveratrol is a natural product, widely available in grapes, easy to extract, and inexpensive [105]. It can act on 8 out of 10 hub genes, which is the largest number of hub genes among the 20 alternatives, while the remaining three alternatives act on only 2 hub genes. Resveratrol has been shown to inhibit VZV virus and IS protection in experiments [18, 106–117].

The recent studies have also found a protective effect of resveratrol in older females [107], with the main mechanism being the protection of older female IS patients through the estrogen pathway [108]. This suggests that resveratrol is a natural, alternative drug to estrogen. Resveratrol also reduces the levels of MMPs [109] and improves the integrity of the BBB, which is destroyed for multiple reasons [110–112]. Resveratrol also regulates intestinal flora [113], increases T regulatory cells [114], and possesses vascular endothelial protection [115], antioxidative stress [116], anti-inflammation [117], hypoglycemic effect [118], hypotensive effect [119], hypolipidemic effect [120], vasodilator [121], antiplatelet aggregation [122], anticoagulation [123], antiatherosclerosis [124], and other functions. Together, these protective functions may reduce the incidence of IS after VZV. No resveratrol-related adverse effects and toxicity were reported in volunteers after oral administration of 500 mg/day of resveratrol [125]. Moreover, resveratrol is safe and well tolerated at doses up to 5 g/d and there is no increase in toxicity with long-term intake [126, 127]. Although polyphenols (resveratrol) are generally safe, there is still a need to be aware that higher doses and prolonged use may cause gastrointestinal adverse effects, such as upset stomach, muscle relaxation and sedation [128].

Melatonin, a hormone secreted by the pineal gland in response to photoperiodic responses, has shown promising results in the treatment of several diseases, including IS and VZV [129, 130]. Recent studies have found it to have excellent antioxidative stress, anti-inflammatory and antiviral effects [131]. Hence, melatonin is also an alternative to VZV-IS drugs. Menthol also has therapeutic effects on VZV and IS [132, 133]. Menthol is poor water soluble and prone to be side effects when taken orally, and is more suitable as a topical agent that can exert analgesic, antipruritic and antiviral effects on herpes on the skin surface [134]. Aspirin, the well-known nonsteroidal anti-inflammatory drug, has a variety of effects such as anti-inflammatory and anticoagulant effects [135]. Previous studies have found that aspirin can inhibit VZV activity [136], and aspirin is one of the drugs used for secondary prevention of IS. However, aspirin has the disadvantage of inducing asthma and upper gastrointestinal bleeding. It can be used as an adjuvant therapy for patients without contraindications.

It is unclear whether the atherosclerotic changes result from a linear progression due to ongoing damage triggered by chronic infection with VZV or from periodic endothelial damage and incomplete healing due to reactivation of chronic infection to acutely activated infection. One view is that residual vascular inflammatory changes following an acute infection episode may persist and be further exacerbated by reactivation of new infection stimuli, and subsequent acute infections produce further damage to previously damaged and incompletely repaired vessel walls until this process develops into mature atherosclerotic lesions [137]. However, regardless of the mechanism, anti-inflammatory, antiviral, antioxidative and anti-atherosclerotic effects are important therapies and prevention of VZV-IS. Because these mechanisms are involved in the onset and development of IS. Resveratrol is certainly a good choice. To validate the role of resveratrol and hub genes, molecular docking and 100 ns molecular dynamics simulations were performed. The results of resveratrol and hub genes with molecular docking and molecular dynamics simulations showed good binding activity and stable interaction between resveratrol and hub genes. It is suggested that resveratrol may have the potential to play a therapeutic and protective role in VZV-induced IS.

## Conclusion

In terms of sequencing, there are no studies on VZV-IS. We have found the potential mechanism of VZV-IS by identifying DEGs between VZV and IS datasets. KEGG and GO enrichment results include regulation of inflammation and oxidative stress response, regulation of vascular morphology and function, regulation of extracellular matrix, regulation of ion transport, regulation of cell adhesion. Resveratrol is reasonably suggested as a drug because it is obtained by identifying hub genes, and it is a promising drug to become a therapeutic and preventive agent for VZV-IS. However, there is still no reliable animal model capable of simulating the VZV-IS pathological process. Simple animal models of VZV-infected middle cerebral artery occlusion do not representative of human VZV-IS pathological processes. Future development of stable animal models of VZV-IS is the basis for exploring the molecular mechanisms of VZV-IS and pharmacological experiments.

### Supplementary Information

**Additional file 1**. The specific information of 20 potential drugs for the treatment and prevention of VZV-IS.

**Additional file 2**. Molecular docking information for VZV-IS drugs and their potential targets.

## Data Availability

The original contributions presented in the study are included in the article, further inquiries can be directed to the corresponding authors.

## Abbreviations

BP: Biological process
CC: Cellular component
DEGs: Differentially expressed genes
ECM: Extracellular matrix
GO: Gene ontology
HDL-C: High-density lipoprotein cholesterol
HIV: Human immunodeficiency virus
IS: Ischemic stroke
KEGG: Kyoto Encyclopedia Genes Genomes
LDL: Low-density lipoprotein
MMPs: Matrix metalloproteinases
MD: Molecular dynamics
MF: Molecular function
NO: Nitric oxide
PHN: Postherpetic neuralgia
PPI: Protein–protein interaction
RMSF: Root mean square fluctuation
RMSD: Root-mean-square deviation
TF: Transcription factor
VZV: Varicella zoster virus

## References

[1] Carcel C, Woodward M, Wang X, Bushnell C, Sandset EC (2020). Sex matters in stroke: a review of recent evidence on the differences between women and men. Front Neuroendocrinol.

[2] Virani SS, Alonso A, Benjamin EJ, Bittencourt MS, Callaway CW, Carson AP, Chamberlain AM, Chang ALR, Cheng SS, Delling FN, Djousse L, Elkind MSV, Ferguson JF, Fornage M, Khan SS, Kissela BM, Knutson KL, Kwan TW, Lackland DT, Lewis TT, Lichtman JH, Longenecker CT, Loop MS, Lutsey PL, Martin SS, Matsushita K, Moran AE, Mussolino ME, Perak AM, Rosamond WD, Roth GA, Sampson UKA, Satou GM, Schroeder EB, Shah STH, Shay CM, Spartano NL, Stokes A, Tirschwell DL, VanWagner LB, Tsao CW (2020). Heart disease and stroke statistics-2020 update: a report from the American Heart Association. Circulation.

[3] Ning XJ, Sun J, Jiang RC, Lu HY, Bai LL, Shi M, Tu J, Wu YA, Wang JH, Zhang JN (2017). Increased stroke burdens among the low-income young and middle aged in Rural China. Stroke.

[4] Hathidara MY, Saini V, Malik AM (2019). Stroke in the young: a global update. Curr Neurol Neurosci Rep.

[5] Lin HC, Chien CW, Ho JD (2010). Herpes zoster ophthalmicus and the risk of stroke: a population-based follow-up study. Neurology.

[6] Zhou JL, Li J, Ma LL, Cao S (2020). Zoster sine herpete: a review. Korean J Pain.

[7] Nagel MA, Gilden D (2016). Developments in varicella zoster virus vasculopathy. Curr Neurol Neurosci Rep.

[8] Amlie-Lefond C, Gilden D (2016). Varicella zoster virus: a common cause of stroke in children and adults. J Stroke Cerebrovasc Dis.

[9] Vangiliappan K, Venkatraman C, Samivel B, Ranganathan LN, Govindarajan S (2019). A study on neurological manifestations of primary varicella zoster virus infection. Neurology Asia.

[10] Yang F, Yu SY, Fan BF, Liu YQ, Chen YX, Kudel I, Concialdi K, DiBonaventura M, Hopps M, Hlavacek P, Cappelleri JC, Sadosky A, Parsons B, Udall M (2019). The epidemiology of herpes zoster and postherpetic neuralgia in China: results from a cross-sectional study. Pain Ther.

[11] Wang W, Pan DQ, Cheng T, Zhu H (2022). Rational design of a skin- and neuro-attenuated live varicella vaccine: a review and future perspectives. Viruses-Basel.

[12] Hambleton S (2005). Chickenpox. Curr Opin Infect Dis.

[13] Oliver SL, Zhou MM, Arvin AM (2020). Varicella-zoster virus: molecular controls of cell fusion-dependent pathogenesis. Biochem Soc Trans.

[14] Gialloreti LE, Merito M, Pezzotti P, Naldi L, Gatti A, Beillat M, Serradell L, di Marzo R, Volpi A (2010). Epidemiology and economic burden of herpes zoster and post-herpetic neuralgia in Italy: a retrospective, population-based study. BMC Infect Dis.

[15] Singh N, Agrawal M, Dore S (2013). Neuroprotective properties and mechanisms of resveratrol in in vitro and in vivo experimental cerebral stroke models. ACS Chem Neurosci.

[16] Shimazu R, Anada M, Miyaguchi A, Nomi Y, Matsumoto H (2021). Evaluation of blood−brain barrier permeability of polyphenols, anthocyanins, and their metabolites. J Agric Food Chem.

[17] Liu JY, He JL, Huang Y, Hu ZP (2021). Resveratrol has an overall neuroprotective role in ischemic stroke: a meta-analysis in rodents. Front Pharmacol.

[18] Docherty JJ, Sweet TJ, Bailey E, Faith SA, Booth T (2006). Resveratrol inhibition of varicella-zoster virus replication in vitro. Antiviral Res.

[19] Safran M, Rosen N, Twik M, BarShir R, Stein TI, Dahary D, Fishilevich S, Lancet D, Abugessaisa I, Kasukawa T (2021). The GeneCards suite. Practical Guide to Life Science Databases.

[20] Amberger J, Hamosh A (2017). Searching Online Mendelian inheritance in man (OMIM): a knowledgebase of human genes and genetic phenotypes. CP Bioinform.

[21] Bubak AN, Como CN, Hassell JE, Mescher T, Frietze SE, Niemeyer CS, Cohrs RJ, Nagel MA (2022). Targeted RNA sequencing of VZV-infected brain vascular adventitial fibroblasts indicates that amyloid may be involved in VZV vasculopathy. Neurol-Neuroimmunol Neuroinflamm.

[22] Thul PJ, Lindskog C (2018). The human protein atlas: a spatial map of the human proteome. Protein Sci.

[23] Zhou YY, Zhou B, Pache L, Chang M, Khodabakhshi AH, Tanaseichuk O, Benner C, Chanda SK (2019). Metascape provides a biologist-oriented resource for the analysis of systems-level datasets. Nat Commun.

[24] Zeng P, Su HF, Ye CY, Qiu SW, Tian Q (2021). Therapeutic mechanism and key alkaloids of *Uncaria rhynchophylla* in Alzheimer's disease from the perspective of pathophysiological processes. Front Pharmacol.

[25] Szklarczyk D, Gable AL, Nastou KC, Lyon D, Kirsch R, Pyysalo S, Doncheva NT, Legeay M, Fang T, Bork P, Jensen LJ, von Mering C (2021). The STRING database in 2021: customizable protein–protein networks, and functional characterization of user-uploaded gene/measurement sets. Nucleic Acids Res.

[26] Doncheva NT, Morris JH, Gorodkin J, Jensen LJ (2019). Cytoscape StringApp: network analysis and visualization of proteomics data. J Proteome Res.

[27] Chin CH, Chen SH, Wu HH, Ho CW, Ko MT, Lin CY (2014). cytoHubba: identifying hub objects and sub-networks from complex interactome. BMC Syst Biol.

[28] Zhou GY, Soufan O, Ewald J, Hancock REW, Basu N, Xia JG (2019). NetworkAnalyst 3.0: a visual analytics platform for comprehensive gene expression profiling and meta-analysis. Nucleic Acids Res.

[29] Davis AP, Wiegers TC, Johnson RJ, Sciaky D, Wiegers J, Mattingly CJ (2023). Comparative Toxicogenomics Database (CTD): update 2023. Nucleic Acids Res.

[30] Kim S, Thiessen PA, Bolton EE, Chen J, Fu G, Gindulyte A, Han LY, He JE, He SQ, Shoemaker BA, Wang JY, Yu B, Zhang J, Bryant SH (2016). PubChem substance and compound databases. Nucleic Acids Res.

[31] Daina A, Michielin O, Zoete V (2019). Swiss Target Prediction: updated data and new features for efficient prediction of protein targets of small molecules. Nucleic Acids Res.

[32] Banerjee P, Eckert AO, Schrey AK, Preissner R (2018). ProTox-II: a webserver for the prediction of toxicity of chemicals. Nucleic Acids Res.

[33] Burley SK, Berman HM, Christie C, Duarte JM, Feng ZK, Westbrook J, Young J, Zardecki C (2018). RCSB Protein Data Bank: sustaining a living digital data resource that enables breakthroughs in scientific research and biomedical education. Protein Sci.

[34] Wang X, Li J, Liu L, Kan J-M, Niu P, Yu Z-Q, Ma C, Dong F, Han M-X, Li J, Zhao D-X (2022). Pharmacological mechanism and therapeutic efficacy of Icariside II in the treatment of acute ischemic stroke: a systematic review and network pharmacological analysis. BMC Complement Med Ther.

[35] Lee J, Cheng X, Swails JM, Yeom MS, Eastman PK, Lemkul JA, Wei S, Buckner J, Jeong JC, Qi YF, Jo S, Pande VS, Case DA, Brooks CL, MacKerell AD, Klauda JB, Im W (2016). CHARMM-GUI input generator for NAMD, GROMACS, AMBER, OpenMM, and CHARMM/OpenMM simulations using the CHARMM36 additive force field. J Chem Theory Comput.

[36] Batista PR, Wilter A, Durham E, Pascutti PG (2006). Molecular dynamics simulations applied to the study of subtypes of HIV-1 protease common to Brazil, Africa, and Asia. Cell Biochem Biophys.

[37] Jorgensen WL, Chandrasekhar J, Madura JD, Impey RW, Klein ML (1983). Comparison of simple potential functions for simulating liquid water. J Chem Phys.

[38] Maier JA, Martinez C, Kasavajhala K, Wickstrom L, Hauser KE, Simmerling C (2015). ff14SB: improving the accuracy of protein side chain and backbone parameters from ff99SB. J Chem Theory Comput.

[39] Van der Spoel D, Lindahl E, Hess B, Groenhof G, Mark AE, Berendsen HJC (2005). GROMACS: fast, flexible, and free. J Comput Chem.

[40] Wang X, Zhao DX, Kan JM, Wang J, Chen X, Yu ZQ, Zhao WS, Han MX, Li JH (2022). Uncovering the mechanism of Chuanhong stroke capsule in the treatment of stroke based on network pharmacology and molecular docking technology. Nat Prod Commun.

[41] Bakradze E, Esenwa CC, Schmid DS, Kirchoff-Torres KF, Antoniello D, Mabie PC, Labovitz DL, Miao CR, Liberman AL (2022). Cross-sectional retrospective study to identify clinical and radiographic features associated with VZV reactivation in cryptogenic stroke patients with CSF testing. Neurohospitalist.

[42] Wu HF, Li JS, Wang RR, Chen C, Hou XJ, Bi XY (2022). A case of ischemic stroke secondary to varicella-zoster virus meningoencephalitis. J Neurovirol.

[43] Lindahl K, Rubin CJ, Brandstrom H, Karlsson MK, Holmberg A, Ohlsson C, Mellstrom D, Orwoll E, Mallmin H, Kindmark A, Ljunggren O (2009). Heterozygosity for a coding SNP in COL1A2 confers a lower BMD and an increased stroke risk. Biochem Biophys Res Commun.

[44] Rajan AM, Ma RC, Kocha KM, Zhang DJ, Huang P (2020). Dual function of perivascular fibroblasts in vascular stabilization in zebrafish. PLoS Genet.

[45] Liu W, Pang B, Lu M, Song H, Sun BM, Zhu YF, Pang Q (2012). The rs42524 COL1A2 polymorphism is associated with primary intracerebral hemorrhage in a Chinese population. J Clin Neurosci.

[46] Shen Y, Russo V, Zeglinski MR, Sellers SL, Wu ZG, Oram C, Santacruz S, Merkulova Y, Turner C, Tauh K, Zhao HY, Bozin T, Bohunek L, Zeng HS, Seidman MA, Bleackley RC, McManus BM, Ruoslahti E, Jarvinen TAH, Granville DJ (2017). Recombinant decorin fusion protein attenuates murine abdominal aortic aneurysm formation and rupture. Sci Rep.

[47] Xu YZ, Zhao KJ, Yang ZG, Zhang YH, Zhang YW, Hong B, Liu JM (2012). Decreased plasma decorin levels following acute ischemic stroke: correlation with MMP-2 and differential expression in TOAST subtypes. Mol Med Rep.

[48] Foster A, Chalot B, Antoniadi T, Schaefer E, Keelagher R, Ryan G, Thomas Q, Philippe C, Bruel AL, Sorlin A, Thauvin-Robinet C, Bardou M, Luu M, Quenardelle V, Wolff V, Woodley J, Vabres P, Lim D, Igbokwe R, Joseph A, Walker H, Jester A, Ellenbogen J, Johnson D, Rooke B, Moss C, Cole T, Faivre L (2020). Kosaki overgrowth syndrome: a novel pathogenic variant in PDGFRB and expansion of the phenotype including cerebrovascular complications. Clin Genet.

[49] Shibahara T, Ago T, Nakamura K, Tachibana M, Yoshikawa Y, Komori M, Yamanaka K, Wakisaka Y, Kitazono T (2020). Pericyte-mediated tissue repair through PDGFR beta promotes peri-infarct astrogliosis, oligodendrogenesis, and functional recovery after acute ischemic stroke. Eneuro.

[50] Cuoco JA, Busch CM, Klein BJ, Benko MJ, Stein R, Nicholson AD, Marvin EA (2018). ACTA2 cerebral arteriopathy: not just a puff of smoke. Cerebrovasc Dis.

[51] Guo DC, Papke CL, Tran-Fadulu V, Regalado ES, Avidan N, Johnson RJ, Kim DH, Pannu H, Willing MC, Sparks E, Pyeritz RE, Singh MN, Dalman RL, Grotta JC, Marian AJ, Boerwinkle EA, Frazier LQ, LeMaire SA, Coselli JS, Estrera AL, Safi HJ, Veeraraghavan S, Muzny DM, Wheeler DA, Willerson JT, Yu RK, Shete SS, Scherer SE, Raman CS, Buja LM, Milewicz DM (2009). Mutations in Smooth Muscle Alpha-Actin (ACTA2) cause coronary artery disease, stroke, and moyamoya disease, along with thoracic aortic disease. Am J Hum Genet.

[52] Kim S, Lee W, Jo H, Sonn SK, Jeong SJ, Seo S, Suh J, Jin J, Kweon HY, Kim TK, Moon SH, Jeon S, Kim JW, Kim YR, Lee EW, Shin HK, Park SH, Oh GT (2022). The antioxidant enzyme Peroxiredoxin-1 controls stroke-associated microglia against acute ischemic stroke. Redox Biol.

[53] Liu Q, Zhang Y (2019). PRDX1 enhances cerebral ischemia−reperfusion injury through activation of TLR4-regulated inflammation and apoptosis. Biochem Biophys Res Commun.

[54] Kisucka J, Chauhan AK, Patten IS, Yesilaltay A, Neumann C, Van Etten RA, Krieger M, Wagner DD (2008). Peroxiredoxin1 prevents excessive endothelial activation and early atherosclerosis. Circ Res.

[55] Wang FC, Yu ZL, Rong K (2020). Correlation of serum GFAP, PRDX1 NPT with severity and prognosis of cerebral ischemic stroke. Acta Medica Mediterranea.

[56] Baerts L, Brouns R, Kehoe K, Verkerk R, Engelborghs S, De Deyn P, Hendriks D, De Meester I (2017). Acute ischemic stroke severity, progression, and outcome relate to changes in dipeptidyl peptidase IV and fibroblast activation protein activity. Transl Stroke Res.

[57] Ohashi M, Runge MS, Faraci FM, Heistad DD (2006). MnSOD deficiency increases endothelial dysfunction in ApoE-deficient mice. Arterioscler Thromb Vasc Biol.

[58] Jung JE, Kim GS, Chen H, Maier CM, Narasimhan P, Song YS, Niizuma K, Katsu M, Okami N, Yoshioka H, Sakata H, Goeders CE, Chan PH (2010). Reperfusion and neurovascular dysfunction in stroke: from basic mechanisms to potential strategies for neuroprotection. Mol Neurobiol.

[59] Mendis DB, Ivy GO, Brown IR (1998). SPARC/osteonectin mRNA is induced in blood vessels following injury to the adult rat cerebral cortex. Neurochem Res.

[60] Ciceri P, Elli F, Cappelletti L, Tosi D, Savi F, Bulfamante G, Cozzolino M (2016). Osteonectin (SPARC) expression in vascular calcification: in vitro and ex vivo studies. Calcif Tissue Int.

[61] Zhou Y, Peng J, Cheng LM, Peng Y, Zhang MM, Liu M, Avery J, Zhou JB, Jiang YG (2019). Secreted Protein Acidic and Cysteine Rich (SPARC) regulates the pathological response to ischemic insults and represents a promising therapeutic target for stroke treatment. Adv Therap.

[62] Becker HM, Rullo J, Chen M, Ghazarian M, Bak S, Xiao HY, Hay JB, Cybulsky MI (2013). alpha 1 beta 1 integrin-mediated adhesion inhibits macrophage exit from a peripheral inflammatory lesion. J Immunol.

[63] Gu C, Zhang HJ, Gao Y (2021). Adipose mesenchymal stem cells-secreted extracellular vesicles containing microRNA-192 delays diabetic retinopathy by targeting ITGA1. J Cell Physiol.

[64] Row S, Liu YY, Alimperti S, Agarwal SK, Andreadis ST (2016). Cadherin-11 is a novel regulator of extracellular matrix synthesis and tissue mechanics. J Cell Sci.

[65] Bowler MA, Bersi MR, Ryzhova LM, Jerrell RJ, Parekh A, Merryman WD (2018). Cadherin-11 as a regulator of valve myofibroblast mechanobiology. Am J Physiol Heart Circul Physiol.

[66] Monahan TS, Andersen ND, Panossian H, Kalish JA, Daniel S, Shrikhande GV, Ferran C, LoGerfo FW (2007). A novel function for cadherin 11/osteoblast-cadherin in vascular smooth muscle cells: modulation of cell migration and proliferation. J Vasc Surg.

[67] Alimperti S, You H, George T, Agarwal SK, Andreadis ST (2014). Cadherin-11 regulates both mesenchymal stem cell differentiation into smooth muscle cells and the development of contractile function in vivo. J Cell Sci.

[68] Du Z, Liu JL, You YH, Wang LZ, He J, Zheng JW, Zhang ZY, Wang YA (2021). Genetic landscape of common venous malformations in the head and neck. J Vasc Surg-Venous Lymph Disord.

[69] Raivich G, Behrens A (2006). Role of the AP-1 transcription factor c-Jun in developing, adult and injured brain. Prog Neurobiol.

[70] Caracciolo L, Marosi M, Mazzitelli J, Latifi S, Sano Y, Galvan L, Kawaguchi R, Holley S, Levine MS, Coppola G, Portera-Cailliau C, Silva AJ, Carmichael ST (2018). CREB controls cortical circuit plasticity and functional recovery after stroke. Nat Commun.

[71] Xu PF, Liu Q, Xie Y, Shi XL, Li YZ, Peng MN, Guo HQ, Sun R, Li JJ, Hong Y, Liu XF, Xu GL (2018). Breast cancer susceptibility protein 1 (BRCA1) rescues neurons from cerebral ischemia/reperfusion injury through NRF2-mediated antioxidant pathway. Redox Biol.

[72] Wang YZ, Zhang HY, Liu F, Li L, Deng SM, He ZY (2019). Association between PPARG genetic polymorphisms and ischemic stroke risk in a northern Chinese Han population: a case-control study. Neural Regen Res.

[73] Liang ZX, Wu GL, Fan CX, Xu J, Jiang S, Yan XL, Di SY, Ma ZQ, Hu W, Yang Y (2016). The emerging role of signal transducer and activator of transcription 3 in cerebral ischemic and hemorrhagic stroke. Prog Neurobiol.

[74] Diaz-Canestro C, Reiner MF, Bonetti NR, Liberale L, Merlini M, Wust P, Amstalden H, Briand-Schumacher S, Semerano A, Giacalone G, Sessa M, Beer JH, Akhmedov A, Luscher TF, Camici GG (2019). AP-1 (activated protein-1) transcription factor JunD regulates ischemia/reperfusion brain damage via IL-1 beta (interleukin-1 beta). Stroke.

[75] Weinl C, Vega SC, Riehle H, Stritt C, Calaminus C, Wolburg H, Mauel S, Breithaupt A, Gruber AD, Wasylyk B, Olson EN, Adams RH, Pichler BJ, Nordheim A (2015). Endothelial depletion of murine SRF/MRTF provokes intracerebral hemorrhagic stroke. Proc Natl Acad Sci USA.

[76] He TL, Shang JL, Gao CL, Guan X, Chen YY, Zhu LW, Zhang LY, Zhang CJ, Zhang J, Pang T (2021). A novel SIRT6 activator ameliorates neuroinflammation and ischemic brain injury via EZH2/FOXC1 axis. Acta Pharm Sin B.

[77] Liu S, Jin R, Xiao AY, Zhong W, Li GH (2019). Inhibition of CD147 improves oligodendrogenesis and promotes white matter integrity and functional recovery in mice after ischemic stroke. Brain Behav Immun.

[78] Simi A, Edling Y, Ingelman-Sundberg M, Tindberg N (2005). Activation of c-fos by lipopolysaccharide in glial cells via p38 mitogen-activated protein kinase-dependent activation of serum or cyclic AMP/calcium response element. J Neurochem.

[79] Deng JS, Zhang YR, He GC, Lu HT, Zhao YW, Li YH, Zhu YQ (2021). Arterial wall injury and miRNA expression induced by stent retriever thrombectomy under stenotic conditions in a dog model. J Neurointerv Surg.

[80] Hsieh CC, Yen MH, Yen CH, Lau YT (2001). Oxidized low density lipoprotein induces apoptosis via generation of reactive oxygen species in vascular smooth muscle cells. Cardiovasc Res.

[81] Li JF, Li WY, Su J, Liu WM, Altura BT, Altura BM (2003). Hydrogen peroxide induces apoptosis in cerebral vascular smooth muscle cells: possible relation to neurodegenerative diseases and strokes. Brain Res Bull.

[82] Fouladseresht H, Talepoor AG, Farjadian S, Khosropanah S, Doroudchi M (2019). Anti-varicella zoster virus IgG and hsCRP levels correlate with progression of coronary artery atherosclerosis. Iran J Allergy Asthma Immunol.

[83] Zhu YH, Xian XM, Wang ZZ, Bi YC, Chen QG, Han XF, Tang DQ, Chen RJ (2018). Research progress on the relationship between atherosclerosis and inflammation. Biomolecules.

[84] Nagel MA, Choe A, Rempel A, Wyborny A, Stenmark K, Gilden D (2015). Differential regulation of matrix metalloproteinases in varicella zoster virus-infected human brain vascular adventitial fibroblasts. J Neurol Sci.

[85] Waite AAC, Hamilton DO, Pizzi R, Ageno W, Welters ID (2020). Hypercoagulopathy in severe COVID-19: implications for acute care. Thromb Haemost.

[86] Poor HD (2021). Pulmonary thrombosis and thromboembolism in COVID-19. Chest.

[87] McFadyen JD, Stevens H, Peter K (2020). The emerging threat of (micro)thrombosis in COVID-19 and its therapeutic implications. Circ Res.

[88] Mackey RH, Venkitachalam L, Sutton-Tyrrell K, Safar ME, Frohlich ED (2007). Calcifications, Arterial Stiffness and Atherosclerosis. Atherosclerosis, Large Arteries and Cardiovascular Risk.

[89] Shi X, Gao J, Lv QS, Cai HD, Wang F, Ye RD, Liu XF (2020). Calcification in atherosclerotic plaque vulnerability: friend or foe?. Front Physiol.

[90] Ito M, Watanabe M, Kamiya H, Sakurai M (1996). Inhibition of natural killer (NK) cell activity against varicella-zoster virus (VZV)-infected fibroblasts and lymphocyte activation in response to VZV antigen by nitric oxide-releasing agents. Clin Exp Immunol.

[91] Li A, Banerjee J, Leung CT, Peterson-Yantorno K, Stamer WD, Civan MM (2011). Mechanisms of ATP release, the enabling step in purinergic dynamics. Cell Physiol Biochem.

[92] Su YC, Kondrikov D, Block ER (2005). Cytoskeletal regulation of nitric oxide synthase. Cell Biochem Biophys.

[93] Johnston-Cox HA, Koupenova M, Ravid K (2012). A2 adenosine receptors and vascular pathologies. Arterioscler Thromb Vasc Biol.

[94] Freedman JE, Loscalzo J, Barnard MR, Alpert C, Keaney JF, Michelson AD (1997). Nitric oxide released from activated platelets inhibits platelet recruitment. J Clin Investig.

[95] Freedman JE, Ting B, Hankin B, Loscalzo J, Keaney JF, Vita JA (1998). Impaired platelet production of nitric oxide predicts presence of acute coronary syndromes. Circulation.

[96] Puchhammer-Stockl E, Aberle SW, Heinzl H (2012). Association of age and gender with alphaherpesvirus infections of the central nervous system in the immunocompetent host. J Clin Virol.

[97] Wu SB, Yang SM, Ou MX, Chen JM, Huang JB, Xiong DL, Sun WP, Xiao LZ (2021). Transcriptome analysis reveals the role of cellular calcium disorder in varicella zoster virus-induced post-herpetic neuralgia. Front Mol Neurosci.

[98] Pan SW, Yen YF, Feng JY, Chuang PH, Su VYF, Kou YR, Su WJ, Chan YJ (2020). Opposite effects of statins on the risk of tuberculosis and herpes zoster in patients with diabetes: a population-based cohort study. Br J Clin Pharmacol.

[99] Antoniou T, Zheng H, Singh S, Juurlink DN, Mamdani MM, Gomes T (2014). Statins and the risk of herpes zoster: a population-based cohort study. Clin Infect Dis.

[100] Koupenova M, Kehrel BE, Corkrey HA, Freedman JE (2017). Thrombosis and platelets: an update. Eur Heart J.

[101] Kapoor S, Opneja A, Nayak L (2018). The role of neutrophils in thrombosis. Thromb Res.

[102] Nagel MA, Bubak AN (2018). Varicella zoster virus vasculopathy. J Infect Dis.

[103] Hoshino T, Toi S, Toda K, Uchiyama Y, Yoshizawa H, Iijima M, Shimizu Y, Kitagawa K (2019). Ischemic stroke due to virologically-confirmed varicella zoster virus vasculopathy: a case series. J Stroke Cerebrovasc Dis.

[104] Williams MS, Cushman M, Ouyang P, Heckbert SR, Kalyani RR, Vaidya D (2016). Association of serum sex hormones with hemostatic factors in women on and off hormone therapy: the multiethnic study of atherosclerosis. J Womens Health.

[105] Stervbo U, Vang O, Bonnesen C (2007). A review of the content of the putative chemopreventive phytoalexin resveratrol in red wine. Food Chem.

[106] Sasivimolphan P, Lipipun V, Likhitwitayawuid K, Takemoto M, Pramyothin P, Hattori M, Shiraki K (2009). Inhibitory activity of oxyresveratrol on wild-type and drug-resistant varicella-zoster virus replication in vitro. Antiviral Res.

[107] Jeong SI, Shin JA, Cho S, Kim HW, Lee JY, Kang JL, Park EM (2016). Resveratrol attenuates peripheral and brain inflammation and reduces ischemic brain injury in aged female mice. Neurobiol Aging.

[108] Shin JA, Oh S, Ahn JH, Park EM (2015). Estrogen receptor-mediated resveratrol actions on blood−brain barrier of ovariectomized mice. Neurobiol Aging.

[109] Dong WP, Gao DK, Lin H, Zhang X, Li NL, Li FF (2008). New insights into mechanism for the effect of resveratrol preconditioning against cerebral ischemic stroke: possible role of matrix metalloprotease-9. Med Hypotheses.

[110] Yang RC, Lv YJ, Miao L, Zhang HP, Qu XY, Chen JQ, Xu BJ, Yang B, Fu JY, Tan C, Chen HC, Wang XR (2021). Resveratrol attenuates meningitic *Escherichia coli*-mediated blood−brain barrier disruption. ACS Infect Dis.

[111] Wei HD, Wang SQ, Zhen LM, Yang QZ, Wu ZX, Lei XM, Lv JR, Xiong LZ, Xue RL (2015). Resveratrol attenuates the blood−brain barrier dysfunction by regulation of the MMP-9/TIMP-1 balance after cerebral ischemia reperfusion in rats. J Mol Neurosci.

[112] Chang HC, Tai YT, Cherng YG, Lin JW, Liu SH, Chen TL, Chen RM (2014). Resveratrol attenuates high-fat diet-induced disruption of the blood−brain barrier and protects brain neurons from apoptotic insults. J Agric Food Chem.

[113] Dou ZC, Rong XF, Zhao EX, Zhang LX, Lv YQ (2019). Neuroprotection of resveratrol against focal cerebral ischemia/reperfusion injury in mice through a mechanism targeting gut-brain axis. Cell Mol Neurobiol.

[114] Yang HN, Zhang AX, Zhang YQ, Ma S, Wang CL (2016). Resveratrol pretreatment protected against cerebral ischemia/reperfusion injury in rats via expansion of T regulatory cells. J Stroke Cerebrovasc Dis.

[115] Clark D, Tuor UI, Thompson R, Institoris A, Kulynych A, Zhang X, Kinniburgh DW, Bari F, Busija DW, Barber PA (2012). Protection against recurrent stroke with resveratrol: endothelial protection. PLoS ONE.

[116] Lin MC, Liu CC, Lin YC, Liao CS (2021). Resveratrol protects against cerebral ischemic injury via restraining lipid peroxidation, transition elements, and toxic metal levels, but enhancing anti-oxidant activity. Antioxidants.

[117] Zhang HF, Zhao WJ (2022). Resveratrol alleviates ischemic brain injury by inhibiting the activation of pro-inflammatory microglia via the CD147/MMP-9 pathway. J Stroke Cerebrovasc Dis.

[118] Szkudelski T, Szkudelska K (2015). Resveratrol and diabetes: from animal to human studies. BBA-Mol Basis Dis.

[119] Bhatt SR, Lokhandwala MF, Banday AA (2011). Resveratrol prevents endothelial nitric oxide synthase uncoupling and attenuates development of hypertension in spontaneously hypertensive rats. Eur J Pharmacol.

[120] Xie HC, Han HP, Chen Z, He JP (2014). A study on the effect of resveratrol on lipid metabolism in hyperlipidemic mice. Afr J Tradit Complement Altern Med.

[121] Emamat H, Djafarian K, Tangestani H, Hekmatdoost A, Shab-Bidar S (2019). Resveratrol supplementation and flow-mediated dilation: a systematic review. Nutr Food Sci.

[122] Bigdeli M, Sabbaghan M, Esfahanizadeh M, Kobarfard F, Vitalini S, Iriti M, Sharifi-Rad J (2019). Synthesis of imine congeners of resveratrol and evaluation of their anti-platelet activity. Molbank.

[123] Chiba T, Kimura Y, Suzuki S, Tatefuji T, Umegaki K (2016). Trans-resveratrol enhances the anticoagulant activity of warfarin in a mouse model. J Atheroscler Thromb.

[124] Mattison JA, Wang MY, Bernier M, Zhang J, Park SS, Maudsley S, An SS, Santhanam L, Martin B, Faulkner S, Morrell C, Baur JA, Peshkin L, Sosnowska D, Csiszar A, Herbert RL, Tilmont EM, Ungvari Z, Pearson KJ, Lakatta EG, de Cabo R (2014). Resveratrol prevents high fat/sucrose diet-induced central arterial wall inflammation and stiffening in nonhuman primates. Cell Metab.

[125] Sergides C, Chirila M, Silvestro L, Pitta D, Pittas A (2016). Bioavailability and safety study of resveratrol 500 mg tablets in healthy male and female volunteers. Exp Ther Med.

[126] Edwards JA, Beck M, Riegger C, Bausch J. In: Vang O, Das DK, editors. Resveratrol and health. 2011;1215:131–7. 10.1111/j.1749-6632.2010.05855.x 21261651

[127] Patel KR, Scott E, Brown VA, Gescher AJ, Steward WP, Brown K. In Vang O, Das DK, editors. Resveratrol and Health. 2011;1215:161–9. 10.1111/j.1749-6632.2010.05853.x 21261655

[128] Wang X, Li JJ, Zhao DX, Li JH (2022). |Therapeutic and preventive effects of apigenin in cerebral ischemia: a review. Food Funct.

[129] Yawoot N, Govitrapong P, Tocharus C, Tocharus J (2021). Ischemic stroke, obesity, and the anti-inflammatory role of melatonin. BioFactors.

[130] Nunes OD, Pereira RD (2008). Regression of herpes viral infection symptoms using melatonin and SB-73: comparison with Acyclovir. J Pineal Res.

[131] Ghaleh HEG, Hosseini A, Aghamollaei H, Fasihi-Ramandi M, Alishiri G, Saeedi-Boroujeni A, Hassanpour K, Mahmoudian-Sani MR, Farnoosh G (2022). NLRP3 inflammasome activation and oxidative stress status in the mild and moderate SARS-CoV-2 infected patients: impact of melatonin as a medicinal supplement. Zeitschrift Fur Naturforschung Section C-a J Biosci.

[132] Yucharoen R, Meepowpan P, Tragoolpua Y (2012). Inhibitory Effect of Peppermint Extracts and Menthol against Herpes Simplex Virus Infection. Chiang Mai J Sci.

[133] Huang SS, Su HH, Chien SY, Chung HY, Luo ST, Chu YT, Wang YH, MacDonald IJ, Lee HH, Chen YH (2022). Activation of peripheral TRPM8 mitigates ischemic stroke by topically applied menthol. J Neuroinflamm.

[134] Liu BY, Fan L, Balakrishna S, Sui AW, Morris JB, Jordt SE (2013). TRPM8 is the principal mediator of menthol-induced analgesia of acute and inflammatory pain. Pain.

[135] Newton JL (2006). Improving the gastrointestinal tolerability of aspirin in older people. Clin Interv Aging.

[136] Primache V, Binda S, De Benedittis G, Barbi M (1998). In vitro activity of acetylsalicylic acid on replication of varicella-zoster virus. Microbiologica.

[137] Liuba P, Persson J, Luoma J, Yla-Herttuala S, Pesonen E (2003). Acute infections in children are accompanied by oxidative modification of LDL and decrease of HDL cholesterol, and are followed by thickening of carotid intima-media. Eur Heart J.

