# Supplementary material for: Resveratrol: a potential medication for the prevention and treatment of varicella zoster virus-induced ischemic stroke
Source: Eur J Med Res. 2023 Oct 5;28:400. doi: 10.1186/s40001-023-01291-4 (PMC10552394; doi:10.1186/s40001-023-01291-4)
Supplement: Supplementary file 2 — Additional file 2. Molecular docking information for VZV-IS drugs and their potential targets. [file 40001_2023_1291_MOESM2_ESM.docx]

Supplementary Table S2. Molecular docking information for VZV-IS drugs and their potential targets.

| Targets | PDB /APSD ID | Drug | Box center (x, y, z) | Affinity/(kcal/mol) |
| --- | --- | --- | --- | --- |
| ACTA2 | AF-P62736-F1 | Melatonin | 0.225, 1.098, 0.906 | -5.28 |
| SOD2 | 2ADP | Melatonin | -8.677, 36.635, 215.905 | -5.23 |
| PRDX1 | 3HY2 | Menthol | 0.085, -7.798, 7.77 | -6.37 |
| SOD2 | 2ADP | Menthol | -8.677, 36.635, 215.905 | -5.23 |
| SPARC | 1SRA | Aspirin | 40.203, 30.460, 32.281 | -4.93 |
| SOD2 | 2ADP | Aspirin | -15.827, 35.140, 231.088 | -4.19 |
